# Metatranscriptomic analysis of the gut microbiome of black soldier fly larvae reared on lignocellulose-rich fiber diets unveils key lignocellulolytic enzymes

**DOI:** 10.3389/fmicb.2023.1120224

**Published:** 2023-04-26

**Authors:** Eric G. Kariuki, Caleb Kibet, Juan C. Paredes, Gerald Mboowa, Oscar Mwaura, John Njogu, Daniel Masiga, Timothy D. H. Bugg, Chrysantus M. Tanga

**Affiliations:** ^1^International Centre of Insect Physiology and Ecology (icipe), Nairobi, Kenya; ^2^Department of Immunology and Molecular Biology, Makerere University, Kampala, Uganda; ^3^Department of Chemistry, School of Life Sciences, University of Warwick, Coventry, United Kingdom

**Keywords:** *Hermetia illucens*, RNA-Sequencing, substrate specificity, bioprospecting, bacterial metabolism

## Abstract

Recently, interest in the black soldier fly larvae (BSFL) gut microbiome has received increased attention primarily due to their role in waste bioconversion. However, there is a lack of information on the positive effect on the activities of the gut microbiomes and enzymes (CAZyme families) acting on lignocellulose. In this study, BSFL were subjected to lignocellulose-rich diets: chicken feed (*CF*), chicken manure (CM), brewers’ spent grain (BSG), and water hyacinth (WH). The mRNA libraries were prepared, and RNA-Sequencing was conducted using the PCR-cDNA approach through the MinION sequencing platform. Our results demonstrated that BSFL reared on BSG and WH had the highest abundance of *Bacteroides* and *Dysgonomonas*. The presence of GH51 and GH43_16 enzyme families in the gut of BSFL with both α-L-arabinofuranosidases and exo-alpha-L-arabinofuranosidase 2 were common in the BSFL reared on the highly lignocellulosic WH and BSG diets. Gene clusters that encode hemicellulolytic arabinofuranosidases in the CAZy family GH51 were also identified. These findings provide novel insight into the shift of gut microbiomes and the potential role of BSFL in the bioconversion of various highly lignocellulosic diets to fermentable sugars for subsequent value-added products (bioethanol). Further research on the role of these enzymes to improve existing technologies and their biotechnological applications is crucial.

## 1. Introduction

There has been a recent need for the development, commercialization, and use of eco-friendly renewable fuels as cleaner alternatives to the rapidly depleting petroleum-based (fossil) fuels. Since first-generation biofuels are produced from food crops such as corn and sugarcane, the dynamic has shifted to second-generation biofuels produced from non-edible cellulosic- and lignocellulosic-rich feedstocks (Limayem and Ricke, 2012), which have no impact on food prices (Robak and Balcerek, 2018). Pretreatment is required to break down the lignin and to disrupt cellulose’s crystalline structure so that the enzymes can easily access and hydrolyze the cellulose and hemicellulose into fermentable sugars. The high costs incurred in the pretreatment and hydrolysis of lignocellulosic feedstocks are not incurred in first-generation biofuel production. Therefore, optimizing the production of second-generation biofuels to compete with petrochemical fuels and first-generation biofuels economically is required (Xiros et al., 2013). Hence, an urgent need is to find inexpensive and time-efficient ways to pretreat and hydrolyze lignocellulosic biomass (Antunes et al., 2019).

Most plant-feeding insects are unable to utilize lignocellulosic biomass as their main sources of nutrition (Sun and Zhou, 2011). However, cellulosic degradation has been observed in about 78 species and more than eight distinct insect orders, including Diptera where the black soldier fly (*Hermetia illucens* L) is classified (Martin, 1991). The black soldier fly larvae (BSFL) are potential lignocellulosic biomass degraders since they are highly polyphagous – capable of consuming large and diverse quantities of organic wastes attributable to their powerful mouthparts and digestive enzymes (Bonelli et al., 2020). Nevertheless, wood-feeding termites and beetles are the most efficient cellulose degraders with up to 99% diet assimilation efficiencies in the former (Martin, 1991).

The BSFL gut microbiome, thought to be involved in bioconversion, has emerged as an active area of study (Samayoa et al., 2016). The BSFL are a useful tool in the valorization of organic biomass and other biodegradable wastes due to their broad diet degradation capabilities and fast growth rates (Sheppard et al., 2002; Nguyen et al., 2015; Klammsteiner et al., 2020). Furthermore, dietary interventions alter the gut microbiome in BSFL; desired microbial communities can be induced through chan dietary regimens (Rothe and Blaut, 2013; Bruno et al., 2019; Klammsteiner et al., 2020).

Members of the genera *Dysgonomonas, Bacteroides*, and *Actinomycetes* with lignocellulolytic capabilities have been identified in high abundance in BSFL (Jeon et al., 2011; Jiang et al., 2019; Tanga et al., 2021). Classical microbial profiling methods rely on targeted amplicon sequencing of the highly conserved marker regions (Langille et al., 2013), while shotgun metagenomics can identify microbial communities, genes, and their potential functions using DNA sequences at high resolution; these techniques do not elucidate the active functions of bacterial species (Chung et al., 2020). Alternatively, metatranscriptomics provides detailed insights into the active functions of the microbial community, and their relationships with the host (Martinez et al., 2016).

Nonetheless, metatranscriptomics focuses on functionally active microbes and genes in a microbiome of interest and is hence more reliable as it filters out the noise – the inability to differentiate live from dead microbiota (Shakya et al., 2019; Qian et al., 2020). The enzymes can be annotated from active genes using databases such as the CAZy database,^1^ which contains the unique classification and curation of Carbohydrate Active enZymes (CAZymes) (Busk et al., 2017). CAZymes comprise all enzymes involved in the biosynthesis and modification of carbohydrates and their derivatives intracellularly and extracellularly. These enzymes have found great use in biotechnology within sectors including but not limited to bioenergy, textile, and other bio-based industries (Contesini et al., 2021).

Bioprocesses have shown high dependence on polysaccharide-degrading CAZymes for deconstructing lignocellulosic fractions of plant biomass during their transformation into value-added products such as biofuels and animal feeds. The identification of lignocellulose-metabolizing microbial communities, coupled with the curation and accurate prediction of CAZyme functions has become a fundamental step in industrial bioprocesses. CAZymes used in biofuel production are mainly obtained from genetically engineered organism strains or commercially produced (Tingley et al., 2021). CAZymes work together to break down recalcitrant carbohydrates effusively and the genes that encode these proteins have a tendency to form gene clusters that are physically linked (Ausland et al., 2021) known as polysaccharide utilization loci (PULs) (Terrapon et al., 2018).

By sequencing the metatranscriptome, i.e., mRNA of the BSFL microbiome, this data was used to study the microbial and functional shifts under different dietary regimens and their possible contribution to lignocellulolytic activity (Li et al., 2019). By implementing the metatranscriptomics pipeline, the active microbial species were identified by annotation against the RefSeq bacterial database and the SEED subsystems hierarchical database, and the respective functions performed by these microbes are inferred (Westreich et al., 2018). This study uses metatranscriptomics to identify and functionally characterize microorganisms involved in the degradation of lignocellulosic biomass in the BSFL gut microbiota and identify microbial enzymes that break down recalcitrant biopolymers.

## 2. Materials and methods

### 2.1. Study site

The study was conducted at the International Center for Insect Physiology and Ecology (*icipe*), Duduville campus, Kasarani, Nairobi, Kenya, where the BSFL were sourced, reared, and RNA extraction and sequencing conducted at the Molecular Biology and Bioinformatics Unit (MBBU) laboratories under the Insects for Food, Feed and Other Uses (INSEFF) program.

### 2.2. Black soldier fly larvae (BSFL) breeding and colony maintenance

One-day old neonates (larvae) were carefully placed in five different diets (2 kg per diet per tray) selected based on their lignocellulosic fiber content. The diets included, chicken manure (CM), brewers’ spent grain (BSG), water hyacinth (WH), processed chicken feed (*CF*) (i.e., control diets) and feed mix (FM). The feed mix (FM) consisted of equal proportions of the various diets (CM, *CF*, BSG, and WH). One kilogram of respective diets were mixed with distilled water in the ratio 2:3 and added in the rearing trays every 5 days until the end of the larval phase of the experiment (Heussler et al., 2019). The rearing conditions of BSFL were maintained at 27°C, 70 ± 5% humidity, and a photoperiod of 12 h:12 h in a greenhouse structure. Each trial was replicated three times.

### 2.3. Sampling and physicochemical parameter collection

At day 14, when the larvae were sizeable (2nd – 3rd instars), 20 larvae were sampled per diet for length and weight measurements. Other parameters such as color, substrate reduction index, pH and temperature of the frass, and the pupation duration and rate of BSFL per diet were also recorded.

### 2.4. Feed composition analysis

The nutrient composition of the various diets used to rear the BSFL were submitted to Crop Nutrition Laboratory Services Limited (Cropnuts Ltd), Limuru, Kenya. Complete feed composition analysis was carried out using the wet chemistry Inductive Coupled Plasma – Mass Spectrometry technique (Wetchem, ICP-MS).

### 2.5. RNA isolation

At the 5th instar stage of the larval development, 15 BSFL were randomly selected and the entire guts dissected. The dissection was conducted by removing the entire gut cavity through the anal opening by gently pulling it out using a pair of forceps. The dissected guts were stored in RNALater^™^ at -80°C. The stored gut samples were thawed on ice for 1 h then RNA was extracted using the Bioline ISOLATE II RNA Mini Kit from Meridian Bioscience as per the manufacturer’s specifications.

### 2.6. Library preparation and sequencing

RNA isolated from BSFL guts was used to generate cDNA libraries using the PCR Barcoding kit (SQK-PCB109) kit from ONT according to the manufacturer’s instructions. The kit allows for reverse transcription and strand-switching using VN primers and multiplexing up to 12 samples in one sequencing run using unique barcode primers provided with the kit. Total RNA was used as the template for all the samples, which included at least three replicates per dietary condition. Barcodes provided with the library preparation kit were assigned to each of the samples to allow for multiplex sequencing, and two sequencing runs were undertaken (Table 1). When performing PCR amplification to select for full-length cDNA transcripts, the following conditions were used: initial denaturation for 30 s at 95°C, followed by 17 cycles of denaturation for 15 s at 95°C, annealing at 62°C for 15 s, and extension at 65°C for 120–150 s. The final extension was carried out for 6 min at 65°C. The concentration and quality of the cDNA libraries were analyzed using NanoDrop 2000 (Thermofisher) and visually using 1.5% agarose gel was run in Tris/Borate/EDTA (TBE) buffer at 70 volts for 1 h with 3.5 μl of each sample, 2 μl of loading dye, and 1.5 μl of 1 Kb ladder. Gel electrophoresis was carried out to analyze the integrity and size of the resultant cDNA libraries.

**Table 1:** Classification of reads and orientation statistics of black soldier fly larvae reared on various diets after sequencing.

Barcode	Sample ID	Classified reads (%)	Unclassified reads (%)	Rescued reads (%)	Orientation (%)	
					+	–
barcode07_1	CF1	903,473 (85.2)	153,373 (14.8)	60 (0)	389,347 (43.1)	514,126 (56.9)
barcode08_1	FM1	691,444 (85.8)	114,370 (14.2)	62 (0)	317,178 (45.9)	374,266 (54.1)
barcode09_1	BSG1	953,056 (87.7)	133,750 (12.3)	54 (0)	427,421 (45.9)	515,635 (54.1)
barcode10_1	CM1	803,584 (86.6)	124,406 (13.4)	46 (0)	368,384 (45.8)	435,200 (54.2)
barcode11_1	WH1	884,170 (82.9)	182,151 (17.1)	32 (0)	365,074 (41.3)	519,096 (58.7)
barcode01	CM2	170,876 (90.3)	18,343 (9.7)	2 (0)	100,894 (59)	69,982 (41)
barcode02	CF2	56,024 (91)	5,536 (9)	6 (0)	28,455 (50.8)	27,569 (49.2)
barcode03	FM2	96,446 (90.1)	10,643 (9.9)	2 (0)	49,873 (51.7)	46,573 (48.3)
barcode04	WH2	67,176 (89.6)	7,822 (10.4)	12 (0)	33,347 (49.6)	33,829 (50.4)
barcode06	BSG2	181,499 (90.9)	18,109 (9.1)	14 (0)	102,190 (56.3)	79,309 (43.7)
barcode07	CF3	170,007 (91.2)	16,499 (8.8)	6 (0)	92,728 (54.5)	77,279 (45.5)
barcode08	CM3	251,968 (91.2)	24,140 (8.7)	30 (0.1)	133,242 (52.9)	118,726 (47.1)
barcode09	FM3	296,918 (92.2)	24,991 (7.8)	20 (0)	170,296 (57.4)	126,622 (42.6)
barcode10	WH3	170,605 (89.7)	19,584 (10.3)	8 (0)	85,804 (50.3)	84,801 (49.7)
barcode11	BSG3	101,893 (88.6)	13,077 (11.4)	4 (0)	51,286 (50.3)	50,577 (49.7)
barcode12	CF4	144,949 (89.7)	16,625 (10.3)	2 (0)	75,176 (51.9)	69,773 (58.7)

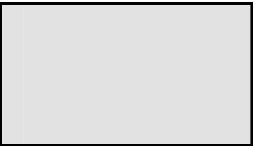
: Sequencing run 1.

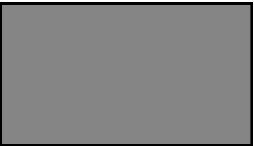
: Sequencing run 2.

Guided by their concentrations, all the amplified barcoded cDNA samples totaling 100 fmol were pooled together to a final volume of 11 μl in Elution Buffer. This was done for both sequencing runs. Before sequencing, 1 μl of sequencing adapters (RAP) were added to the amplified cDNA libraries. Flow cell priming was done using the Flow cell Priming Kit EXP-FLP002 from ONT while adhering to the manufacturer’s specifications. The MinION MK1B sequencing platform was used for both sequencing runs using two types of flow cells were used: R9.4.1 and R10.4.

### 2.7. Bioinformatics analysis

#### 2.7.1. Quality control

The MinKNOW software (version 21.06.0) was used for data acquisition, real-time analysis and feedback, and sample tracking and identification. Basecalling was run on an NVIDIA Tesla V100 Graphical Processing Unit (GPU) with the Guppy software (v5.0.11) using the High-Accuracy Model (HAC) model (Lu et al., 2016) custom command line parameters. The configuration file was modified and the quality score of the reads was set at the minimum threshold of 7. Every read that failed to attain this threshold was discarded.

Once basecalling was complete, sequencing statistics were obtained with the PycoQC tool using the default parameters (Leger and Leonardi, 2019). Pychopper (v2.2.0)^2^ was used to trim orient and defuse chimeric cDNA sequence reads. The *edlib* backend which uses local and global alignment strategies to detect primer hits within the reads was specified alongside default parameters.

Porechop (v0.2.4) was run to remove sequencing adapters using default parameters (Wick et al., 2017). The adapter-free reads were then grouped into gene clusters by running isONclust (v0.0.6.1) with default parameters where each resultant cluster represented a set of reads from a particular gene. To reduce the error rates associated with ONT long-read sequencing, isONcorrect (v0.0.8) was run using the default parameters. *In silico* Ribodepletion using SortMeRNA (v4.3.3) (Kopylova et al., 2012) was performed to remove residual rRNA reads from the corrected reads using default parameters against the silva-arc-16 s-database-id95.fasta, silva-euk-28 s-id98.fasta, silva-euk-18 s-id95.fasta, silva-arc-23 s-id98.fasta, and rfam-5.8 s-database-id98.fasta rRNA databases.

#### 2.7.2. Taxonomic validation using filtered rRNA reads

From the previous ribodepletion step, an assortment of rRNA reads (both eukaryotic and prokaryotic) was obtained. Additional filtering was done using SortMeRNA to retain only the bacterial rRNA sequences (16S rRNA) using the silva-arc-16 s-database-id95.fasta (Quast et al., 2013). A custom pipeline was designed to perform 16S rRNA analysis that involved: (i) merging the sequence files from the same metatranscriptome, (ii) quality filtering and sequence dereplication using Vsearch (v2.16.0) (Rognes et al., 2016) to remove spurious and duplicated sequences, (iii) getting rid of chimeric sequences and generating amplicon sequence variant (ASV) files using Usearch (v11.0) (Edgar and Bateman, 2010), (iv) generating an ASV counts table using the vsearch -usearch_global command at 85% identity, and (v) taxonomic assignment using the Ribosome Database Project (RDP) classifier for 16S rRNA sequences (Cole et al., 2014). The Phyloseq package (v1.24.2) (McMurdie and Holmes, 2013) in Bioconductor (Love et al., 2014) was used in 16S rRNA statistical analysis using modified in-house R Software (v4.0.2) scripts.

#### 2.7.3. Alignment with Minimap2

Minimap2 was used to align the ribodepleted reads to the BSF reference genome downloaded from the National Center for Biotechnology Information (NCBI) genomes database.^3^ Non-default parameters reported by Sahlin and Mäkinen (2021) including options -t 16 -G 500 k -k 13 -w 5 -ax splice were invoked.

#### 2.7.4. Alignment statistics with samtools

SAMtools (v1.12) (Li and Durbin, 2009) was used to obtain mapping statistics for corrected and uncorrected reads. The .sam files generated from alignment with Minimap2 were converted to .bam files before being sorted and indexed. This was followed by running the samtools flagstat command to obtain the percentage of reads mapped, and samtools coverage to obtain the coverage, depth, and mapping quality statistics. The mapping statistics namely coverage, mean depth, and percentage of reads mapped to the reference were used to evaluate the efficacy of the clustering and error correction steps in improving the accuracy and throughput of the sequencing data. A paired *t*-test (*α* = 0.05) was used to check whether the differences between these two groups were statistically significant. The reads that showed better alignment scores were used for the subsequent commands to generate the mapped and unmapped .fastq files for subsequent steps.

#### 2.7.5. Obtaining raw read counts

The number of raw reads in each unmapped sample was computed to facilitate subsequent calculations of diversity and differential expression statistics with DESeq2 (Love et al., 2014). This was done using a Python script (raw_read_counter.py) adopted from the SAMSA2 analysis pipeline (Westreich et al., 2018) using the default parameters.

#### 2.7.6. Annotation of unmapped reads with DIAMOND

The DIAMOND program (Buchfink et al., 2021) was used to annotate our metatranscriptomics datasets against two databases. The NCBI bacterial RefSeq database (Tatusova et al., 2014) was used to generate organism and functional annotations, while the SEED subsystems database was selected to generate annotations for functional activities in a hierarchical format (Overbeek et al., 2014). The databases were indexed in DIAMOND format, and annotation was performed using the BLASTx translated nucleotide search module using the diamond blastx default parameters. The --sensitive parameter was invoked to increase the accuracy of the annotation step.

#### 2.7.7. Aggregation of annotated reads

After annotation, DIAMOND generated results with each sequence from the metatranscriptome with a corresponding hit from the reference database occupying a single line in the annotated output file. From the SAMSA2 pipeline (Westreich et al., 2018), the *standardized_DIAMOND_analysis_counter.py* written in Python language was used to aggregate the RefSeq annotated output files into three-column summarized tables using default parameters. The first column contained the percentage of each entry compared to the total reads, the second column contained the respective read counts, and the third column contained the annotation (organism or function). For hierarchical annotations using the SEED subsystems database, a separate Python script (*DIAMOND_subsystems_analysis_counter.py*) from the SAMSA2 pipeline was used to generate outputs containing information from the different hierarchy levels for later use in R software for statistical analysis and visualization. The output was further compressed using the *DIAMOND_subsystems_reducer.py* script to get the results into a more summarized format for statistical analysis while removing redundant annotations (Westreich et al., 2018).

#### 2.7.8. Statistical analysis and visualization

Statistical analysis was performed using R software (V4.0.2) using in-house scripts adapted from the SAMSA2 pipeline (Westreich et al., 2018). The scripts were utilized to perform within-group and between-group comparisons, normalize raw read counts, calculate Shannon and Simpson diversity statistics, generate stacked taxonomic bar plots, principal component analysis (PCA), and calculate differential expression statistics for the various organisms and functions from DIAMOND annotation results.

#### 2.7.9. CAZymes annotation with dbCAN2 Hotpep module

Metatranscriptome samples from the same diet (4 control samples and 3 from each of the experimental metatranscriptomes) were pooled together and converted into fasta format. CAZymes were annotated by uploading the pooled fasta sequences to the dbCAN2 web server and using the dbCAN standalone software^4^ and screening them against the homology to peptide (Hotpep module) (Busk et al., 2017) which performs annotations by matching the conserved peptides to known protein sequences of interest. The annotation command entailed some non-default parameters specifying the Hotpep module.

#### 2.7.10. Screening for PULs from lignocellulolytic CAZyme families

To further understand whether the highly abundant bacteria identified from our metatranscriptomes were organized as PUL gene clusters responsible for producing lignocellulolytic CAZymes, the dbCAN-PUL BLASTx resource^5^ (Ausland et al., 2021) was used to screen and annotate PULs in the identified CAZy families in comparison with the pooled sequences from the control metatranscriptomes.

**Figure 1:**
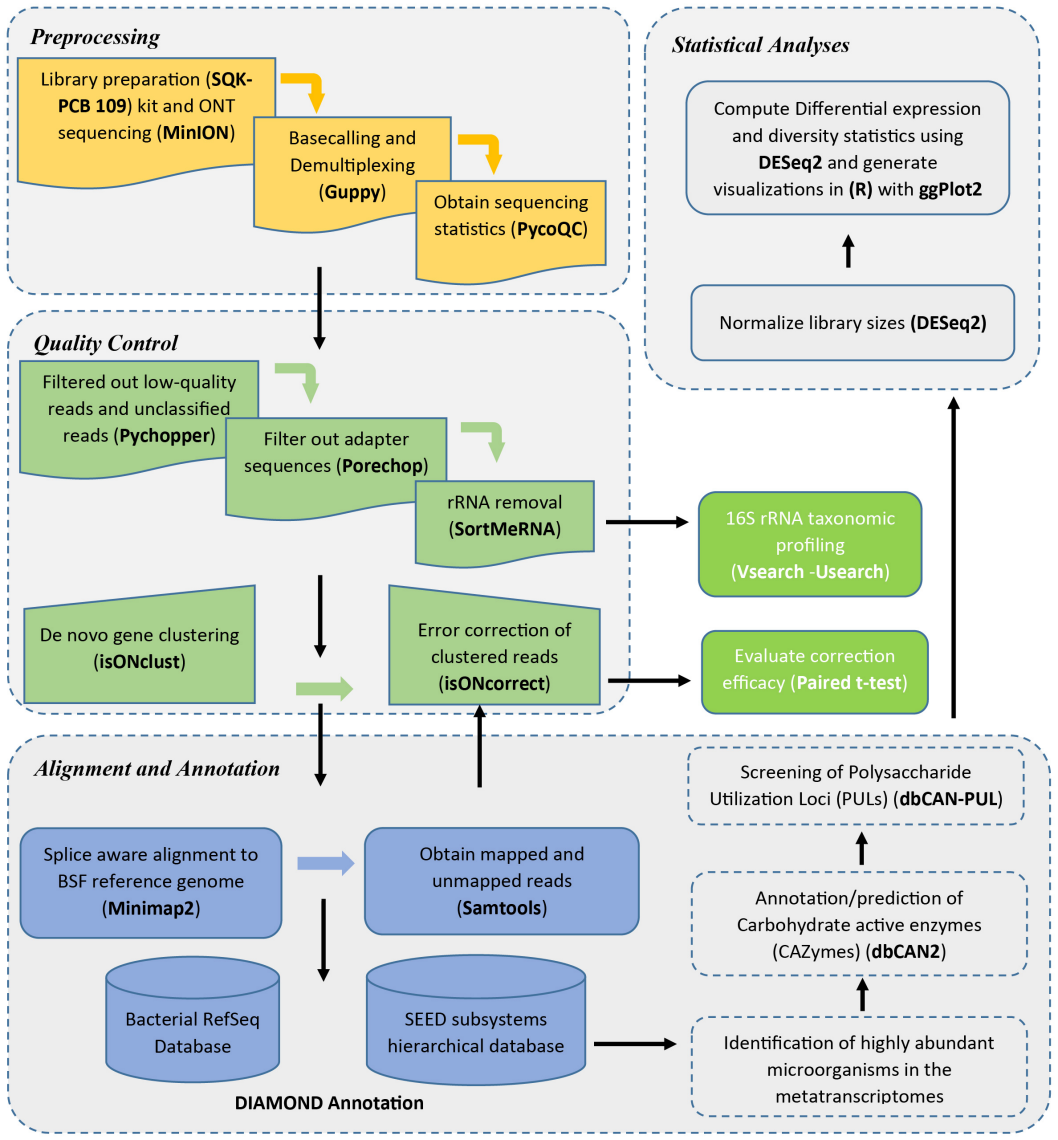
Metatranscriptomics analysis workflow.

Figure 1 shows a detailed workflow of the analysis steps employed in this study.

## 3. Results

### 3.1. Proximate and mineral composition of diets and impact on black soldier fly larvae (BSFL) growth parameters

The nutrient composition of the various diets used in the experiments is shown in Table 2 based on Inductive Coupled Plasma–Mass Spectrometry (ICP-MS) analysis. The protein levels of the diet type varied between 13.98 and 24.1%. The energy content of the chicken feed diet was higher (13 MJ/Kg) compared to the other diets. Water hyacinth (WH) and brewer’s spent grain (BSG) had the highest fiber contents at 23.3 and 19.5%, respectively. The lowest fiber levels (5.95%) were recorded in the control diet, chicken feed (*CF*).

**Table 2:** Proximate and mineral composition of different diets used in rearing the black soldier fly larvae.

Parameter	Method	Unit	Result			
			BSG	*CF*	CM	WH
Energy	Calculated	MJ/Kg	12	13	7.66	9.1
Protein	ISO 5983-2	%	24.1	18.5	19.5	13.98
Total Ash	ISO 5984	%	5.24	6.16	30.2	12.8
Fat	Gafta 3	%	4.43	0.83	1.82	0.915
Fiber	ISO 6865	%	19.5	5.95	17.1	23.3
Dry Matter @103C Animal Feed	ISO6496	%	91.4	89	90.7	89.3
Calcium	CN-TM-P01	%	0.54	0.76	6.06	2.395
Potassium	CN-TM-P01	%	0.12	0.98	1.38	3.475
Magnesium	CN-TM-P01	%	0.25	0.27	0.73	0.24
Phosphorous	CN-TM-P01	%	0.71	0.72	1.36	0.094
Sulfur	CN-TM-P01	%	0.36	0.21	0.3	0.093
Boron	CN-TM-P01	ppm	2.88	8.96	17.4	20.8
Molybdenum	CN-TM-P01	ppm	1.65	1.1	2.57	2.66
Iron	CN-TM-P01	ppm	526	280	2,480	194
Copper	CN-TM-P01	ppm	15.8	16.2	29.4	2.27
Zinc	CN-TM-P01	ppm	149	89.5	259	18.6
Manganese	CN-TM-P01	ppm	65.8	106	591	388.5
Sodium	CN-TM-P01	ppm	150	1,560	2,350	341.75
Cobalt	CN-TM-P01	ppm	0.17	0.63	1.59	0.115

**Figure 2:**
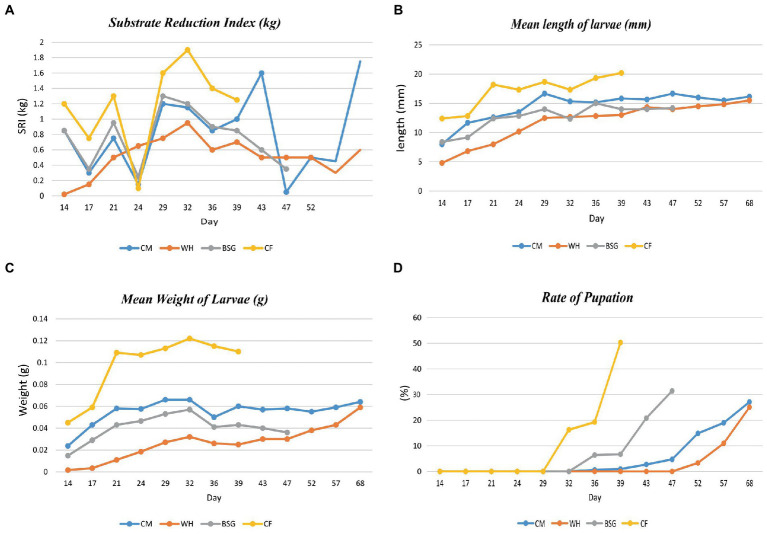
Parameter collection during BSFL rearing. Substrate reduction index **(A)** and growth parameters [Length **(B)**, weight gain **(C)**, and the rate of pupation **(D)**] of BSFL reared on various diets.

Figure 2 provides data on the growth performance of BSFL on the various diets. The highest substrate reduction index, mean length, mean weight, and rate of pupation were recorded when larvae were reared on *CF* compared to the other diets. The developmental time of the BSFL ranged between 39 and 68 days.

### 3.2. Preprocessing and quality control of sequence reads

Table 1 provides the distribution of reads after physical ribodepletion, sequencing, barcode classification, and strand orientation in the various sample replicates for each dietary condition.

The residual rRNA reads filtered from the ribodepletion step varied between 25.6 and 44% of the total reads. The numbers of unmapped reads per sample after reference mapping are indicated in Supplementary Table S1.

**Figure 3:**
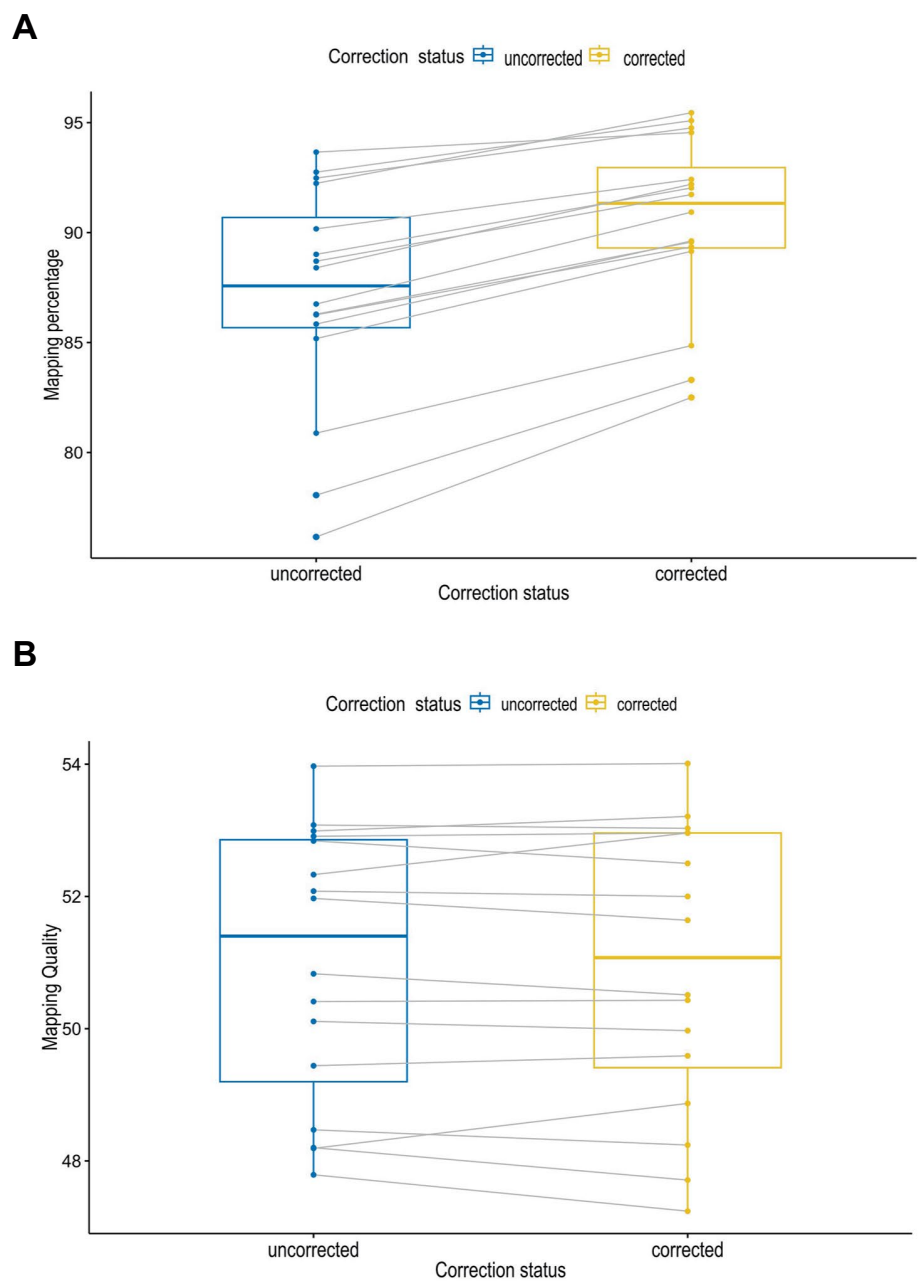
Mapping percentages **(A)** and mapping quality **(B)** of sequence reads before and after correction.

In Figure 3A, the error correction had a significant impact on the accuracy and throughput of the reads as demonstrated by the mapping percentages (*P* = <0.001) at *α* = 0.05. The mapping percentages were higher for the corrected reads as compared to the uncorrected reads. There were no significant differences between the observed mapping quality before and after corrections of reads (*p* = 0.6) (Figure 3B).

### 3.3. Relative activity of microorganisms in individual metatranscriptomes

**Figure 4:**
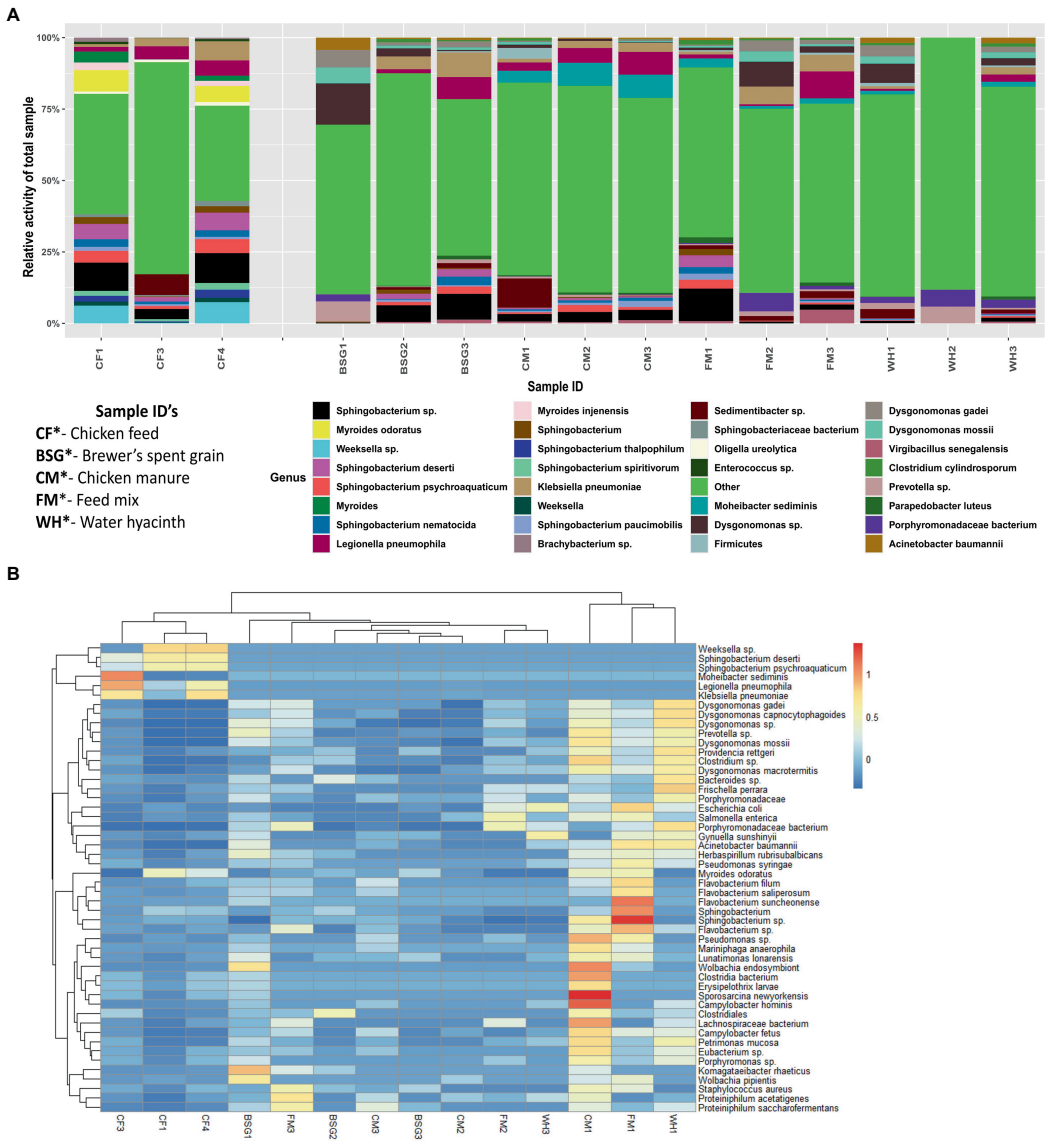
The relative activity **(A)** of various microorganisms and dissimilarity **(B)** between the individual metatranscriptomes.

The relative activity of the microorganisms present in the individual metatranscriptomes revealed notable variations in microbial profiles between the control samples reared on *CF* and experimental samples reared on BSG, CM, FM, and WH diets. *Myroides* sp. and *Weeksella* sp. portrayed high relative activity in the samples reared on *CF*. *Dysgonomonas* sp. (Figures 4A,B) and *Bacteroides* sp. (Figure 4B) showed higher relative activity in the larvae reared on BSG and WH. The larvae reared on FM diet revealed bacteria species such as *Sphingobacterium* sp., *Porphyromodaceae* sp., *Dysgonomonas* sp., and *Legionella* sp. (Figures 4A,B). *Sphingobacterium* sp. had high relative activity in *CF*, BSG, CM, and FM, except on WH (Figures 4A,B). The highest metatranscriptome dissimilarity from the Euclidean distance heatmap (Figure 4B) was observed between *CF* and WH.

### 3.4. Validation of metatranscriptomic analysis using filtered 16S rRNA reads

**Figure 5:**
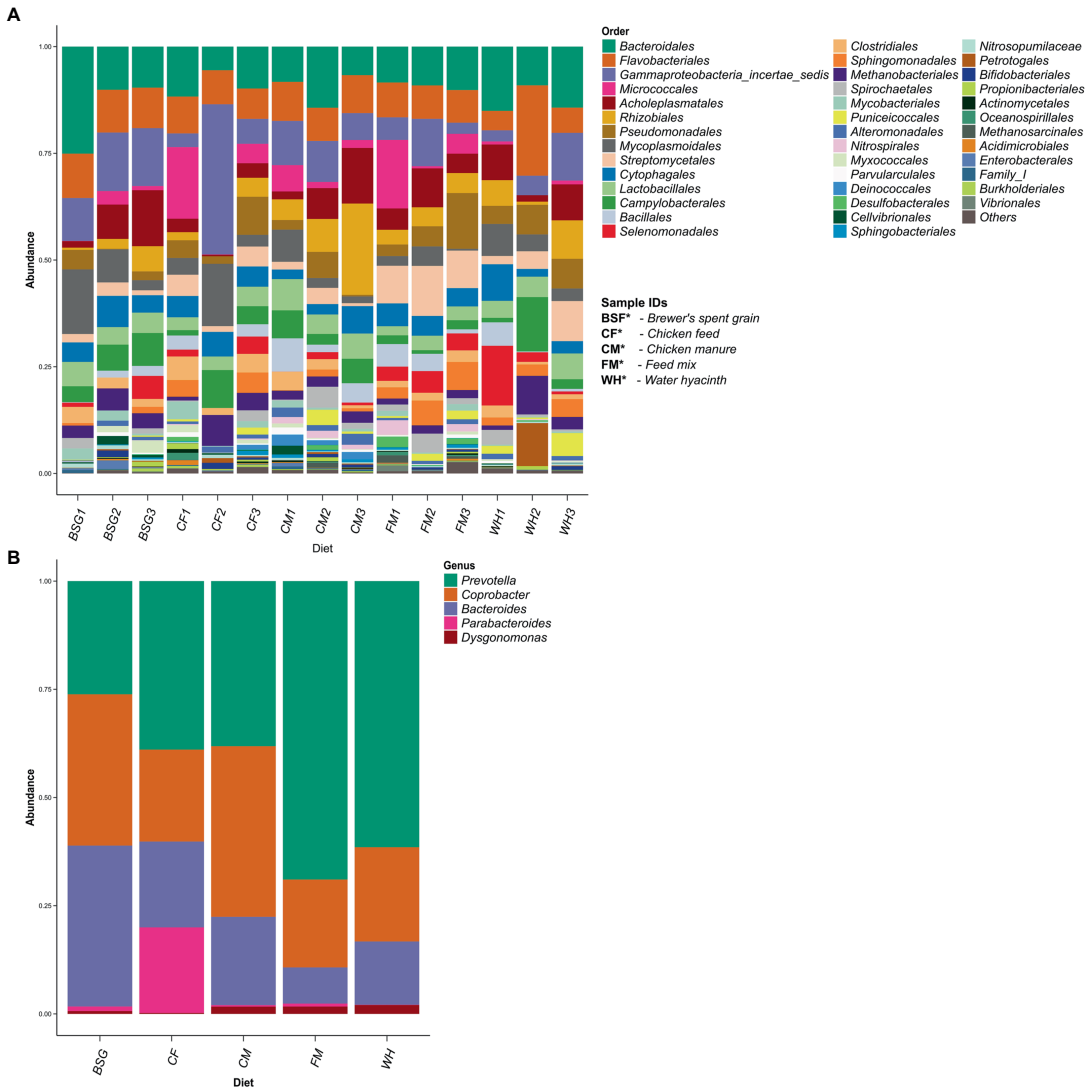
Taxonomic profiling using filtered 16S rRNA sequence reads. Taxonomic orders identified from 16S rRNA profiling **(A)**. A subset of order Bacteroidales – one of the most prevalent orders identified after 16S taxonomic profiling **(B)**.

More than 40 taxonomic orders were identified (Figure 5A). A subset of the order Bacteroidales identified showed 5 dominant bacteria genera: (*Bacteroides, Coprobacter, Dysgonomonas*, *Parabacteroides*, and *Prevotella*). Three of these genera (*Bacteroides, Coprobacter*, and *Prevotella*) were abundant in all the substrates (*CF*, BSG, CM, FM, and WH) (Figure 5B). The relationship between the abundant genera observed is represented on a taxonomic tree shown in Supplementary Figure S2.

### 3.5. Metabolism of abundant bacterial species

**Figure 6:**
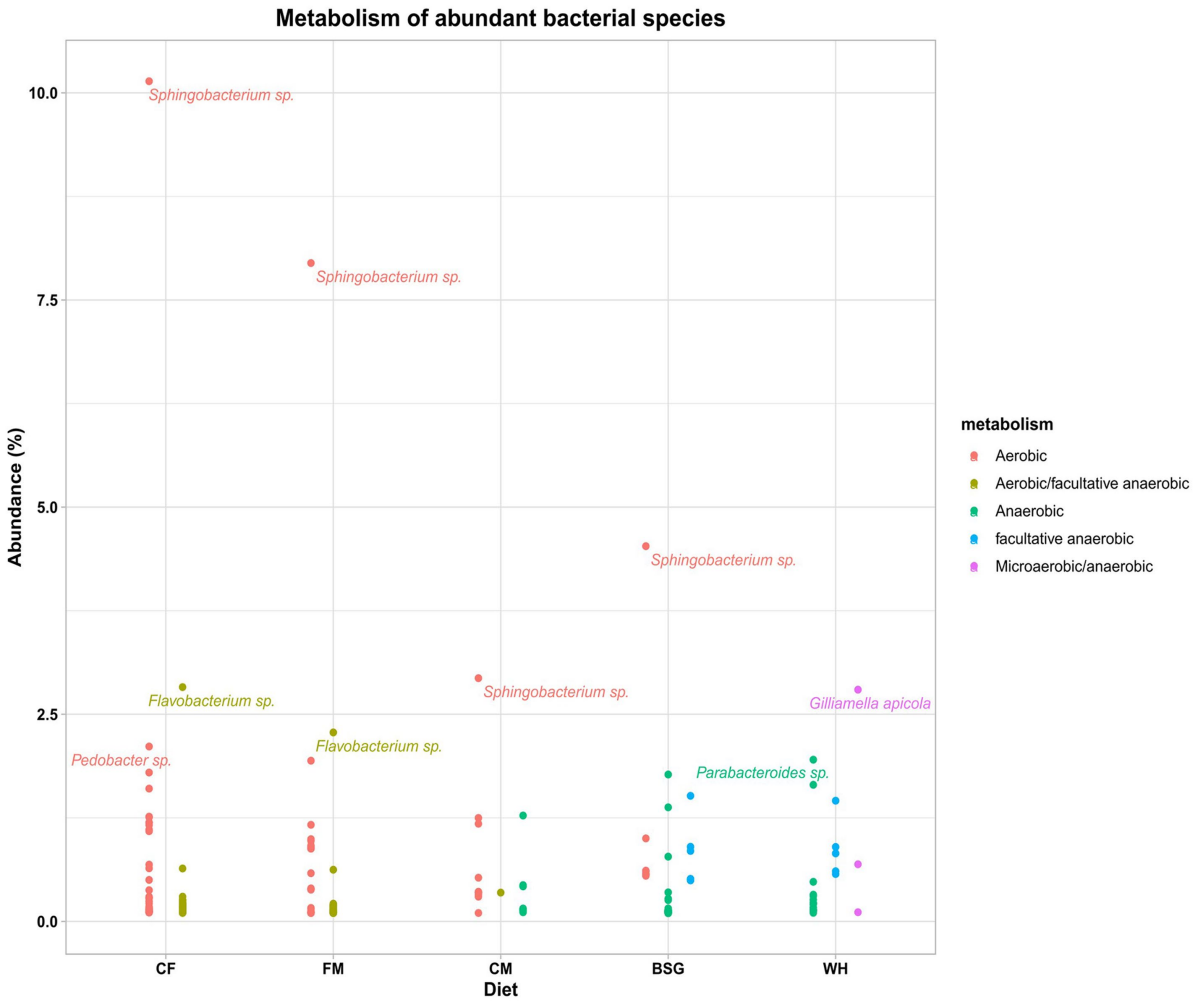
The metabolism (aerobic status) of the abundant bacteria in the metatranscriptomes of the BSFL reared on the various diets – chicken feed (*CF*), feed mix (FM), chicken manure (CM), brewer’s spent grain (BSG), and water hyacinth (WH).

The aerobicity of the BSFL gut was observed to reduce with an increase in fiber content of the various diets (Figure 6). The highest and the lowest aerobicity was recorded when BSFL was fed on *CF* and WH, respectively. The aerobic bacteria *Sphingobacteria* sp. was dominant in all the metatranscriptomes except for that of WH. The metatranscriptomes of WH was dominated by *Gilliamella apicola*, (microaerobic/anaerobic), *Parabacteroides* (anaerobic), *Bacteroides* (anaerobic), and *Dysgonomonas* (facultative anaerobic). However, *Flavobacteria* sp. (aerobic/facultative anaerobic) were more abundant in *CF* metatranscriptomes. Supplementary Table S4 catalogs all bacterial species identified with an abundance of >0.1% from the 4 dominant genera in pooled metatranscriptomes from the various dietary groups.

### 3.6. Metatranscriptome diversity and similarity

**Figure 7:**
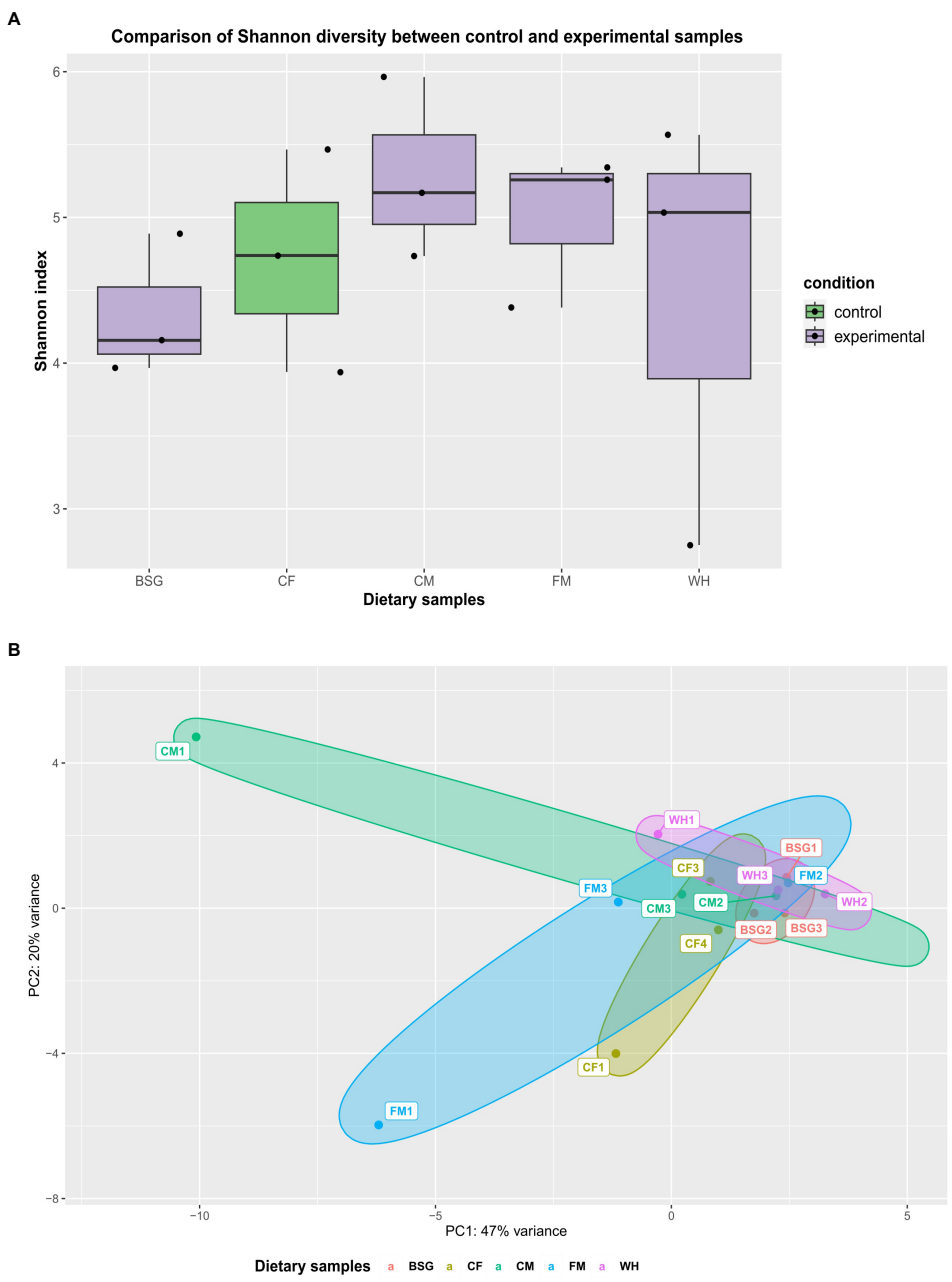
Metatranscriptome diversity and similarity. **(A)** Shannon diversity in pooled and **(B)** Principal Component Analysis (PCA) observed in the individual metatranscriptomes – chicken feed (*CF*), feed mix (FM), chicken manure (CM), brewer’s spent grain (BSG), and water hyacinth (WH).

The Shannon-Wiener index (H) revealed higher species diversity in the metatranscriptomes of BSFL from CM, FM, and WH compared to *CF* (Figure 7A). The two principal components (PC1 and PC2) accounted for 67% of the total variance. The lowest and highest variability of BSFL metatranscriptomes were recorded from BSG and CM, respectively (Figure 7B).

### 3.7. Functional activity in the metatranscriptomes

**Figure 8:**
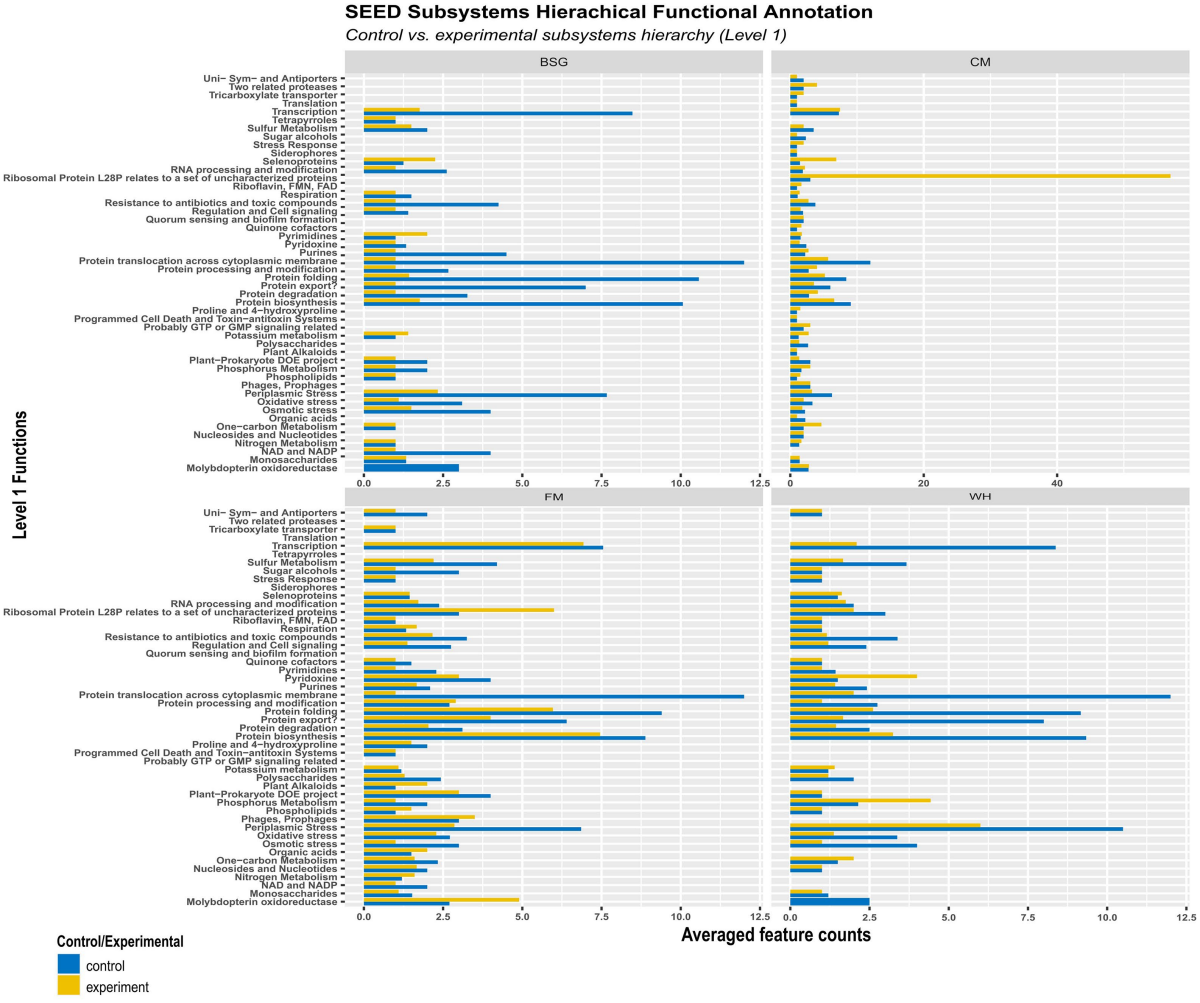
Hierarchical functional annotation of the metatranscriptome groups – chicken feed (*CF*), feed mix (FM), chicken manure (CM), brewer’s spent grain (BSG), and water hyacinth (WH) – using the SEED Subsystems database.

More functional activity was observed in the control (*CF*) than in the experimental metatranscriptomes (BSG, CM, FM, and WH) (Figure 8). The average levels of pyrimidines, potassium metabolism, and selenoproteins observed in the BSG were higher than in *CF*. Transcription, ribosomal protein L28P, selenoproteins, one-carbon metabolism, and RNA processing modification functions were more enriched in BSFL from CM than in *CF*. In metatranscriptomes of BSFL from FM, functions associated with molybdopterin oxidoreductase, phages and prophages, organic acids, and nitrogen metabolism were observed to be higher compared to *CF*. In metatranscriptomes of BSFL from WH, functions associated with pyridoxine and phosphorous metabolism were higher.

### 3.8. CAZyme annotation from metatranscriptone of black soldier fly larvae reared on various diets

Table 3 shows the highest number of CAZymes (11) in the FM metatranscriptomes. GH43_16, CE7 and GH51 were the key lignocellulolytic enzyme groups identified in BSFL reared on BSG, FM and WH, respectively.

**Table 3:** CAZyme screening with dbCAN2 from black soldier fly larvae reared in various diets.

Diet	CAZyme Family (sub-family)	Signature peptides (Hotpep)	Accepted enzyme name and EC number	Reactions catalyzed
*BSG*	GH43(16)	ESRSVT, SRSVTS, EVNRRW, VNRRWT, NRRWTE, RRWTEG	Xylan 1,4-beta-xylosidase (3.2.1.37) Non-reducing end alpha-L-arabinofuranosidase (3.2.1.55) (Mewis et al., 2016)	Hydrolysis of (1- > 4)-beta-D-xylans, to remove successive D-xylose residues from the non-reducing termini (Howard et al., 1960) Hydrolysis of terminal non-reducing alpha-L-arabinofuranoside residues in alpha-L-arabinosides (Tagawa and Kaji, 1969)
	GT2(46)	YILWLD, FAAARN, ILWLDA, LDADDI, WLDADD, LWLDAD, EYILWL		
	GT51(2)	GSTITQ, STITQQ, GGSTIT, QGGSTI, ITQQLA, TITQQL	Peptidoglycan glycosyltransferase (2.4.1.129:58)	(GlcNAc-(1- > 4)-Mur2Ac(oyl-L-Ala-gamma-D-Glu-L-Lys-D-Ala-D-Ala))(n)-diphosphoundecaprenol + GlcNAc-(1- > 4)-Mur2Ac(oyl-L-Ala-gamma-D-Glu-L-Lys-D-Ala-D-Ala)-diphosphoundecaprenol <= > (GlcNAc-(1- > 4)-Mur2Ac(oyl-L-Ala-gamma-D-Glu-L-Lys-D-Ala-D-Ala)) (n + 1)-diphosphoundecaprenol + undecaprenyl diphosphate Aids the biosynthesis of peptidoglycan in bacterial cell walls (Taku et al., 1982)
*CF*	CE11(1)	LDAIGD, MLDAIG, DEFVRH, EFVRHK, HKMLDA, DAIGDL, KMLDAI, MRDIEY, DIEYLQ, RDIEYL, IEYLQS, GFMRDI, FGFMRD, FMRDIE	UDP-3-O-acyl-N-acetylglucosamine deacetylase (3.5.1.108:260)	UDP-3-O-((3R)-3-hydroxyacyl)-N-acetyl-alpha-D-glucosamine + H(2)O < => UDP-3-O-((3R)-3-hydroxyacyl)-alpha-D-glucosamine + acetate The enzyme catalyzes a dedicated step in the biosynthesis of lipid A facilitated by Zn2+ cofactors (Schomburg and Schomburg, 2013)
	CE11(3)	IEIDGP, IDGPEV, PILDGS, GPEVPI, PEVPIL, VPILDG, DGPEVP, EVPILD, EIDGPE		
	CE11(6)	VPDPEN, PGDTLI.,GDTLIF, QTGGIL, AQTGGI, MAQTGG, AMAQTG, EAMAQT, PDPENY		
	GH19(4)	GTNGLA, TNGLAD, NGGTNG, LADRQA, GGTNGL, NGLADR, GLADRQ, INGGTN		
	GH95(1)	PANLQG, QPANLQ, YTININ, KYTINI, FGRYLL, LQGIWN, QFGRYL, NYWPAE, ANLQGI, TININT, GRYLLI, SKYTIN, NLQGIW		
	GT26(29)	IDELPQ, MSLVGP, SLVGPR, DMSLVG, GDMSLV, LVGPRP		
	CBM13(55)	NLQGIW,PANLQG,GRYLLI,QPANLQ,LQGIWN,ANLQGI		
*CM*	GH13(27)	QIFPDR, IFPDRF, FYQIFP, VFYQIF, AVFYQI, YQIFPD	Cyclomaltodextrinase (3.2.1.54:41)	Catalyzes the hydrolysis of cyclodextrin to linear maltodextrin and the hydrolysis of linear maltodextrin in the starch and sucrose metabolism pathway (De Pinto and Leon Campbell, 1968)
*FM*	CE7(2)	FTDAVR, RVFTDA, RRVFTD, TDAVRA, DAVRAV, VFTDAV	Acetylxylan esterase (3.1.1.72:35)	Deacetylation of xylans and xylooligosaccharides. Catalyzes the hydrolysis of acetyl groups from polymeric xylan, acetylated xylose, acetylated glucose, alpha-naphthyl acetate, p-nitrophenyl acetate (Poutanen et al., 1990)
	CE9(1)	TAPAGA, VTDATA, TDATAP, LVTDAT, ATAPAG, DATAPA	N-acetylglucosamine-6-phosphate deacetylase (3.5.1.25:306)	Galactose metabolism, amino and nucleotide sugar metabolism. Catalyzes N-acetyl-D-glucosamine 6-phosphate + H(2)O < => D-glucosamine 6-phosphate + acetate (White and Pasternak, 1967)
	GH0(99)	GCGGRG, VGCGGR, RGTGAA, GGRGTG, GRGTGA, CGGRGT, LVGCGG		
	GH28(11)	GHGGFV, TRGRGG, KSTRGR, FKSTRG, STRGRG, HGGFVV, GGFVVG		
	GH31(13)	AFAGQQ,TRSAFA,KRVFIL,GQQRYG,VFILTR,AGQQRY,RVFILT,RSAFAG,SDKRVF,SAFAGQ,ILTRSA,FAGQQR,LTRSAF,FILTRS		
	GH33(43)	TEAEWE, AEWEYA, PTEAEW, LPTEAE, RLPTEA, EAEWEY		
	GT2(55)	NEKENI, DGSPDG, TYNEKE, IPTYNE, DDGSPD, YNEKEN, SPDGTA, PTYNEK, IIPTYN, LVIIPT, GSPDGT, VIIPTY	Dolichyl-phosphate beta-D-mannosyltransferase (2.4.1.83:53)	Involved in the N-glycan biosynthesis pathway where it catalyzes GDP-mannose + dolichyl phosphate <= > GDP + dolichyl D-mannosyl phosphate(Babczinski et al., 1980)
	GT28(13)	VNPLLA, LAGGGT, PLLATA, TAGHVN, AGGGTA, HVNPLL, GTAGHV, AGHVNP, GGTAGH, LLAGGG, GGGTAG, GHVNPL		
	GT35(1)	GTGNMK, GTLDGA, ASGTGN, LTIGTL, EASGTG, SGTGNM, TIGTLD, GALTIG, ALTIGT, NGALTI, IGTLDG	Glycogen phosphorylase (2.4.1.1:1499)	Entails several enzymes that act *in vivo* on different forms of (1- > 4)-alpha-D-glucans in starch and sucrose metabolism. Known to catalyze the first step in the degradation of large branched glycan polymers - the phosphorolytic cleavage of alpha-1,4-glucosidic bonds from the non-reducing ends of linear poly(1- > 4)-alpha-D-glucosyl chains within the polymers. The reaction is terminated when it reaches the fourth residue away from an alpha-1,6 branching point, leaving a highly branched core known as a limit dextrin (Chen and Segel, 1968)
	CBM50(9)	KLHFEI,VKLHFE,LHFEIR,TGTDRV,TDRVKL,HFEIRR,DRVKLH,GTDRVK,RVKLHF		
	CBM6(53)	TEAEWE, AEWEYA, PTEAEW, EWEYAA, RLPTEA, LPTEAE, EAEWEY		
*WH*	GH0(16)	GRGTGA, LIGCGG, GCGGRG, ALIGCG, GGRGTG, IGCGGR, CGGRGT		
	GH51(2)	GNENWG, WGCGGN, ENWGCG, NENWGC, GCGGNM, NWGCGG	Non-reducing end alpha-L-arabinofuranosidase (3.2.1.55:729)	Hydrolysis of terminal non-reducing alpha-L-arabinofuranoside residues in alpha-L-arabinosides (Tagawa and Kaji, 1969)

### 3.9. Polysaccharide utilization loci (PULs) screening

The range of percentage identity (38.31–92.308%) of PUL hits identified from metatranscriptomes of BSFL reared on BSG and WH varied considerably (Table 4). The top PUL hits (PUL0395, PUL0013, and PUL0309) detected in the high-fiber diet (WH) sequences corresponded to the GH51 CAZy family.

**Table 4:** Polysaccharide utilization loci (PUL) hits identified in black soldier fly larvae reared on high-fiber diets.

Sample ID	PUL ID	Hit name	Hit locus	Hit protein ID	Identity (%)	*E*-value	Annotation
WH	PUL0395	abfB	N.A	ACE73681.1	92.308	1.11E-08	GH51
WH	PUL0013	abfB	N.A	ACE73681.1	92.308	1.11E-08	GH51
WH	PUL0395	abfB	N.A	ACE73681.1	88.889	1.11E-08	GH51
WH	PUL0013	abfB	N.A	ACE73681.1	88.889	1.11E-08	GH51
WH	PUL0309	N.A	*Caldanaerobius polysaccharolyticus/44256*	WP_026486272.1	83.333	1.62E-07	GH51
WH	PUL0309	N.A	*Caldanaerobius polysaccharolyticus/44256*	WP_026486272.1	76.923	1.62E-07	GH51
BSG	PUL0395	abfB	N.A	ACE73681.1	50	2.53E-13	GH51
BSG	PUL0013	abfB	N.A	ACE73681.1	50	2.53E-13	GH51
BSG	PUL0218	N.A	*termite gut metagenome*	CCO20976.1	50	5.14E-11	GH51
BSG	PUL0532	abf2	*Bacteroides cellulosilyticus*	ALJ58197.1	47.368	3.63E-09	GH51
BSG	PUL0218	N.A	*termite gut metagenome*	CCO20976.1	44.737	5.14E-11	GH51
BSG	PUL0190	N.A	*Bacteroides xylanisolvens XB1A*	CBK66679.1	43.182	3.27E-09	GH51
BSG	PUL0302	N.A	*Bacteroides thetaiotaomicron VPI-5482*	AAO75455.1	42.105	3.69E-09	GH51
BSG	PUL0395	abfB	N.A	ACE73681.1	41.509	2.53E-13	GH51
BSG	PUL0013	abfB	N.A	ACE73681.1	41.509	2.53E-13	GH51
BSG	PUL0309	N.A	*Caldanaerobius polysaccharolyticus/44256*	WP_026486272.1	39.623	2.75E-07	GH51
BSG	PUL0302	N.A	*Bacteroides thetaiotaomicron VPI-5482*	AAO75455.1	38.636	3.69E-09	GH51

N.A, Not assigned; abfB, α-L-arabinofuranosidase B; abf2, α-L-arabinofuranosidase 2.

## 4. Discussion

This study demonstrated that dietary intervention using diets of varying lignocellulosic content had a substantial effect on the microorganism profiles and in turn the functional profiles of the BSFL. *Bacteroides* and *Dysgonomonas* genera that were hypothesized at the beginning of this study from preliminary BSFL microbiota studies (Jeon et al., 2011; Jiang et al., 2019; Tanga et al., 2021) to be directly involved in the degradation of lignocellulosic diets were found to be abundant in the two lignocellulosic metatranscriptomes, BSG and WH where two families of α-L-arabinofuranosidases (EC3.2.1.55), GH43 and GH51 with hemicellulolytic capabilities were identified.

As potent as the BSFL are in degrading organic compounds, their waste reduction capabilities show a notable reduction when the diets in question contain recalcitrant compounds such as cellulose and lignin, and in turn, portray altered gut microbial diversity profiles as compared to those observed in processed diets. This has been revealed by diversity analysis using various alpha diversity indices with very small changes being detected at a beta-diversity level (Khamis et al., 2020; Klammsteiner et al., 2020; Tanga et al., 2021). Since metatranscriptomics involves understanding which organisms are performing what functions in a microbial community, the highly active functions were studied by pooling all experimental metatranscriptomes belonging to one diet and comparing them against the pooled control samples: chicken feed (*CF*).

The high digestibility in the control diet (*CF*), was attributed to its low fiber levels (Diener et al., 2009). The BSFL reared on *CF* had the highest substrate reduction index (SRI), mean larval lengths, mean weight of larvae, and the fastest pupation rates, while the opposite pattern was observed on larvae reared on water hyacinth (WH). Based on the inductive coupled plasma-mass spectrometry (ICP-MS) analysis, crude fiber, the dietary fraction that is associated with poorly digestible, recalcitrant components such as lignin, cellulose, and hemicellulose (Van Soest, 1966) was found to be highest in WH (23.3%) and brewer’s spent grain BSG (19.5%) diets. The *CF* diet as a control contained well-balanced nutrients which are readily available and utilizable translating to faster growth and pupation levels (Barragan-Fonseca et al., 2017; Klammsteiner et al., 2020, 2021). However, despite the higher fiber levels recorded in the BSG, larvae reared on this diet attained the threshold pupation rate (25%) faster (day 47) than the CM (day 68) which contained lower fiber levels. This could be explained by the mechanical, biological, and chemical treatments such as milling, heating, mashing, and microbial transformations subjected to BSG during beer production. These transformations reduce the structural integrity of the more recalcitrant components, i.e., lignocellulosic fractions, increasing the levels of readily digestible carbon sources for the larvae (Pérez et al., 2002; Adesogan et al., 2019; Lalander et al., 2019).

Functional analysis showed that most of the functions were highly enriched in the *CF* metatranscriptomes, likely due to the diet’s balanced levels of macronutrients and micronutrients which enhanced its digestibility. Most notably, the *CF* diet contained the highest energy content levels (13 MJ/Kg) and the lowest crude fiber levels (5.95%) providing more available and digestible carbon sources for the larvae, and lower amounts of the slowly digestible long-chain fiber compounds such as lignin, cellulose, and hemicellulose (Pérez et al., 2002). On the other hand, despite considerable energy content levels in the WH diet (9.1 MJ/Kg), the SRI levels, the mean lengths, and the mean weight of the WH-reared larvae were lower than those of the CM diet whose energy content was lower (7.66 MJ/Kg). This observation could be attributed to the high crude fiber levels of the WH diet which render it harder for the larvae to break down due to lower amounts of available digestible carbon (Li et al., 2012; Rehman et al., 2017). A study by Yang et al. (2021) showed that there was a significant reduction in metabolically active microbiota due to starvation or stress which in our case, might be induced by less digestible diets.

From metatranscriptomics analysis, organisms from the genera *Sphingobacterium* were dominant in 4 of the 5 groups (BSG, *CF*, CM, and FM). *Sphingobacteria* sp. have been previously detected in the guts of *Anoplophora glabripennis* beetles that feed on wood diets (Schloss et al., 2006) and have been found to possess xylanases directly involved in the breakdown of hemicellulose fractions (Zhou et al., 2009). The role of *Sphingobacterium* sp. T2 in the microbial breakdown of lignin has also been previously studied (Rashid et al., 2018) where the species was found to produce an extracellular manganese superoxide dismutase, MnSOD1 capable of oxidative demethylation of polymeric lignin.

Further investigation into the metabolism of the most abundant bacteria (species within the top 4 genera with >0.1% abundance) identified in each of the metatranscriptomes revealed that aerobicity in the gut reduced with the increase in the diet’s lignocellulose content. The foregut in most insects is mildly aerobic but rapidly becomes anaerobic further into the midgut and hindgut sections, with more fermentative or anaerobic bacteria species occurring in the hindgut (Kane, 1997). Moreover, the feeding preferences of the BSFL toward less lignocellulosic diets were demonstrated by better growth performance with faster pupation rates, higher mean weights, and mean larval lengths being recorded in the BSFL reared on *CF* that was lower in lignocellulose.

The abundance of aerobic bacteria such as *Sphingobacteria, Flavobacteria, Pedobacter,* and *Myroides* could indicate higher digestive activity in the foregut, especially for the less recalcitrant fractions of the diet. However, the abundance of anaerobic species such as *Bacteroides, Dysgonomonas, Parabacteroides*, and *Prevotella* in the larvae reared on high-fiber diets such as BSG and WH is suggestive of higher lignocellulolytic activity carried out by these species in the midgut and hindgut sections. A study by Bonelli et al. (2020) reported diet-dependent adaptation of the midgut to facilitate the breakdown of non-standard diets. Classically, recalcitrant compounds such as cellulose and hemicellulose are digested by bacteria and symbionts in the hindgut (Brown, 1972; Martin, 1983, 1991). Unlike mammals and wood-feeding insects such as beetles and termites, BSFL are midgut fermenters (Bruno et al., 2019; Seyedalmoosavi et al., 2022). However, aerobic and facultative species have been isolated in the hindgut of some insect species; oxygen diffuses across the hindgut before being rapidly metabolized by the aerobic and facultative species creating a highly anoxic environment for the fermentative anaerobic species (Brune et al., 1995; Kane, 1997).

*Gilliamella* was identified as the most abundant in BSFL reared on WH. This species has well-documented carbohydrate-degrading functions in the honey bee gut microbiota as the principal degrader of hemicellulose and pectin with extensive repertoires of genes associated with the breakdown of polysaccharides (Zheng et al., 2019). In a study by Zhineng et al. (2021), *Gilliamella apicola* was also detected in BSFL reared on wheat bran and soy powder. Nonetheless, it is unclear whether the species perform the same functions in the BSFL microbiome as in the honey bee gut microbiome, though its abundance in a highly lignocellulosic diet might be further indicative of this possibility.

Some classes of bacteria are organized into Polysaccharide Utilization Loci (PULs) clusters to facilitate the breakdown of complex carbohydrates (Ausland et al., 2021). A high abundance of *Sphingobacterium* sp. is associated with a remarkable potential for the degradation of complex polysaccharides (Bruno et al., 2019). However, none of the PULs recorded in the WH samples was associated with bacteria from the Sphingobacteria genera after screening with dbCAN-PUL (Ausland et al., 2021), despite PUL hits associated with CAZy GH51 being reported in some *Sphingobacterium* sp. Also, none of the PULs identified from the dbCAN-PUL and PULDB (Terrapon et al., 2018) databases for CAZy GH43 subfamily 16 found in the BSG sample were associated with *Sphingobacterium* sp. Highly abundant *Sphingobacterium* sp. identified from these metatranscriptomes may not have been organized into polysaccharide-degrading PULs or could be involved in other functions. With PUL resources being recently developed, PULs from *Sphingobacterium* sp. may not have been curated into existing databases.

*Sphingobacterium* species have been identified in some BSFL microbiome studies to constitute the core gut microbiota (Tegtmeier et al., 2021; Yang et al., 2021; Zhineng et al., 2021), but not as the most dominant species. This differed from the results obtained in our study, which showed higher dominance of *Sphingobacterium* sp. in the larvae fed on more digestible diets such as *CF* and CM, an occurrence that needs to be further investigated. However, Rashid et al. (2015, 2018) have reported their involvement in lignin oxidation by the production of extracellular manganese superoxide dismutases.

Genera *Bacteroides* and *Dysgonomonas* belong to the taxonomic order Bacteroidales where roughly 40% of the PULs have been identified, with half of these PULs belonging to the *Bacteroides* genus (Seshadri et al., 2018). PULs PUL0395 and PUL0013 were identified in BSG and WH metatranscriptomes which were rich in the above-mentioned genera but were not associated with any organism clusters in their respective metatranscriptomes. This finding does not rule out the presence of PULs responsible for the breakdown of complex polysaccharides and the production of lignocellulolytic enzymes in our metatranscriptome sequences as the annotations on the dbCAN-PUL database (Ausland et al., 2021) are reliant on published literature. Therefore, novel or unannotated PULs may likely be missing from this resource and subsequent studies should involve biochemical characterization assays to assign such PULs to their respective organisms. The PULs identified from the BSFL reared on the highly lignocellulosic BSG and WH diets (PUL0013 and PUL0395), have been previously isolated from the L-arabinan utilization system of the *Geobacillus stearothermophilus* bacterium, and have been found to possess both CAZy families GH43 and GH51 which were identified in our metatranscriptome sequences. PUL0013 is involved in the degradation of arabinan moieties while PUL0395 is involved in the degradation of arabinan and arabinose (Shulami et al., 2011), both of which make up the hemicellulose heteropolymer (Ahmad and Zakaria2019).

Our samples contained two arabinofuranosidases classes (GH43 and GH51) from the highly lignocellulosic BSG and WH metatranscriptomes, respectively, using the dbCAN2 Hotpep module (Busk et al., 2017). The α-L-arabinofuranosidases (EC3.2.1.55) are exo-enzymes that cleave terminal α-(1,2), α-(1,3), or α-(1,5) linked L-arabinofuranosyl residues from hemicelluloses or oligosaccharides containing arabinose to liberate arabinofuranosides of arabinoxylan, arabinogalactan, and arabinan (McKee et al., 2012). In the CAZy database, these enzymes are classified into families GH2, GH3, GH43, GH51, GH54, and GH62 (Wilkens et al., 2017). GH43 are more specific arabinofuranosidases and act on terminal α-1,5-linked arabinofuranosides while GH51 cleaves both α-1,2 and α-1,3 arabinofuranosyl moieties from xylans and arabinan (Margolles and De los Reyes-Gavilán, 2003). The complete breakdown of hemicellulosic fractions of lignocellulosic biomass can therefore be achieved by the joint activity of these enzyme families. A study conducted by Mewis et al. (2016) that characterized the GH43 enzymes into subfamilies, found carbohydrate-binding module 6 (CBM6) with an established function of binding to β-1,4-xylan and amorphous cellulose, to be highly prevalent in the GH43_16 subfamily which was identified in the BSG metatranscriptome. This subfamily was also found to be multimodular, sharing some protein modules with other GH43 subfamilies potentially broadening its diet specificity.

CAZy class GH0 which was identified in the WH metatranscriptome is annotated as “Not classified” in the CAZy database. This CAZy class possesses enzymes with CAZyme functionality based on significant amino-acid similarity, but await biochemical characterization (Lombard et al., 2014). Therefore, novel CAZy families could be identified from our data upon further biochemical characterization assays. This necessitates subsequent studies aimed at characterizing these novel CAZy families and constant annotation of the extant databases to bridge this knowledge gap.

Metatranscriptomics using Oxford Nanopore Technologies (ONT) long-read sequencing required ribodepletion and error-correction steps. Most ribodepletion kits are optimized for commonly studied hosts, i.e., mammalian, bovine, and murine species, and not invertebrates (Kumar et al., 2016). The SQK-PCB109 kit used during library preparation in this study enriched for mRNA while minimizing rRNA reads. However, a majority of the mRNA reads sequenced were of host origin and had to be filtered out and error correction performed. Reads that could have been otherwise discarded were corrected and retained based on their common regions, regardless of their mean depths. After *de novo* isoform clustering with isONclust (Sahlin and Medvedev, 2020), isONcorrect leverages the shared regions between transcripts by jointly using all the isoforms from a gene, enabling the correction of genes at low sequencing depths (Sahlin et al., 2021).

A sharp reduction in the number of originally classified reads in comparison to the unmapped reads required for the metatranscriptomic organism and functional annotation was observed. The loss of these reads is a common challenge in metatranscriptomic analysis (Shakya et al., 2019). mRNA sequences are a good solution for examining the overall organism prevalence within a microbial community and offer a more comprehensive measure of abundance, diversity, and prevalent functions (Westreich et al., 2016). However, a previous study by Penaranda and Hung (2019) shows that bacterial cells can produce up to 100 times less total mRNA compared to their eukaryotic host. To address the above-mentioned limitations associated with metatranscriptomics analysis, this study utilized a unique approach that combined a non-standard error-correction step with 16S rRNA taxonomic validation using filtered rRNA reads – an approach aimed to improve the reliability and precision of the results.

The reads filtered out during the *in silico* ribodepletion step were used for our rRNA analysis, as done in MetaTrans analysis pipeline (Martinez et al., 2016). From 16S rRNA analysis, the genera *Bacteroides* and *Dysgonomonas* abundant in the lignocellulose-rich BSG and WH metatranscriptomes were also found to be among the most abundant in their respective 16S rRNA sequences. These additional bacterial genera identified from the 16S rRNA analysis, which might represent inactive gut microbiota, were less likely to be identified with metatranscriptomic analysis which focuses only on the functionally active microbes in the community being studied (Bashiardes et al., 2016). The dissimilarities in taxonomic profiles were possible misclassification of highly similar short-length sequences, or sequencing and basecalling errors. Moreover, these filtered sequences targeted no specific variable region of the 16S rRNA gene (Jeong et al., 2021). The 85% identity threshold used on the filtered rRNA reads to empirically validate the results obtained from metatranscriptomics may also have been lenient and unsuitable below the Order taxonomic level (Kaehler et al., 2019). Nonetheless, a subset of the order Bacteroidales revealed genera *Coprobacter* and *Prevotella* as among the five most abundant genera in all diets. However, in metatranscriptomic analysis, genus *Prevotella* was only found to be among the most abundant genera in BSFL reared on the highly lignocellulosic BSG diet, while genus *Coprobacter* was not identified among the four most abundant genera in any of the metatranscriptomes. Findings from different 16S rRNA gene studies have shown some significant differences in the gut microbial composition of BSFL. This has been mainly attributed to the different diets used in each study that are capable of shaping the gut microbial load and diversity profiles (Bruno et al., 2019), and environmental factors credited to the various study sites. The sections of the 16S rRNA gene studied and the choice of analysis tools also contribute to changes in abundance and diversity, especially for lower taxonomic classes (Jeong et al., 2021), but to a lower extent (Chung et al., 2020).

Despite the rising interest and demand for BSFL’s capabilities in bioconversion and as an alternative animal feed, very little has been reported on the larvae’s degradative performance in diets with varying crude fiber content. This study identified and functionally characterized microorganisms with active functions in the BSF larval microbiota using a metatranscriptomics approach while making comparisons with 16S rRNA taxonomic profiling. We further showed the differences in organism and diversity profiles using both metatranscriptomics and 16S rRNA approaches for the BSFL reared with diets of varying fiber content. From this study, a metatranscriptomics analysis pipeline was designed for ONT-cDNA long-read sequences and implemented on reads sequenced from the gut of the BSFL with the ONT MinION MK1B platform. Dietary intervention using diets of varying lignocellulose content was found to induce notable shifts in the microorganism profiles.

## 5. Conclusion

In summary, two of the three genera hypothesized to possess lignocellulolytic abilities, *Dysgonomonas* and *Bacteroides* were found to be among the most dominant in the BSFL fed on the two highly lignocellulosic diets, brewer’s spent grain (BSG) and water hyacinth (WH) from the metatranscriptomic analysis. After subsetting order Bacteroidales where most of the abundant genera from the metatranscriptomic analysis were classified, 16S rRNA analysis using ribodepleted sequences further revealed genera *Bacteroides* and *Dysgonomonas* were among the most abundant species in the highly lignocellulosic diets BSG and WH. Genus *Prevotella* which was identified from metatranscriptomic analysis as among the most abundant in the BSG diet was also identified as among the most abundant from the 16S rRNA analysis. However, genera *Coprobacter* and *Prevotella* identified as the most abundant from the 16S analysis were not among the highly abundant genera in the metatranscriptomic analysis. Since metatranscriptomic analysis only focused on microbial species with active functions in the microbiome, these genera likely represented the inactive gut microbiota. Two families of α-L-arabinofuranosidases (EC3.2.1.55), GH43 and GH51 were identified from the highly lignocellulosic BSG and WH metatranscriptomes. The joint catalytic action of these two enzyme families is capable of the complete hydrolysis of hemicellulosic fractions of lignocellulosic biomass, rendering them potentially useful in biotechnological applications. Therefore, the BSFL gut microbiome potentially presents itself as a source of industrially important microbial communities and enzymes possessing lignocellulolytic activity that could be applied in, but not limited to, enzymatic hydrolysis in second-generation biofuel production and other bioprocesses.

From this study, since metagenomics and targeted sequencing approaches, e.g., 16S rRNA (demonstrated in this study) also reveal inactive microbes in complex microbial communities, we propose metatranscriptomic analyses as the first line of screening for active microbes and enzymes present in these communities before culturing, biochemical characterization assays, and metabolic engineering of strains and enzymes used in various biotechnological applications. Additionally, the abundant filtered host mRNA reads should be utilized in subsequent studies to understand the host-microbial interactions and the effects of dietary intervention on the host gene expression.

## Data availability statement

The datasets presented in this study can be found in online repositories. The names of the repository/repositories and accession number(s) can be found below: GenBank Sequence Read Archive (SRA) under the Accession ID: PRJNA866094.

## Ethics statement

This study was reviewed and approved by Makerere University School of Biomedical Sciences IRB (SBS 2021-63) and the National Council for Science Technology and Innovation in Kenya (NACOSTI/P/21/14306). This research also received approval from the Institutional Animal Care and Use Committee (IACUC) of Kenya Agricultural and Livestock Research Organization (KALRO)-Veterinary Science Research Institute (VSRI); Muguga North upon compliance with all provisions vetted under and coded: KALRO-VSRI/IACUC028/16032022.

## Author contributions

EK: conceptualization, methodology, investigation, data curation, formal analysis, visualization, writing – original draft, and writing – review and editing. JP, CK, and GM: conceptualization, methodology, writing – review and editing, and supervision. DM: supervision, project administration, and writing – review and editing. OM and JN: data curation, formal analysis, visualization, and writing – review and editing. TB: conceptualization, methodology, and writing – review and editing. CT: conceptualization, methodology, investigation, supervision, project administration, and funding acquisition. All authors contributed to the article and approved the submitted version.

## Funding

Financial support for this research was provided by the Australian Centre for International Agricultural Research (ACIAR) (ProteinAfrica—Grant No. LS/2020/154), Horizon Europe (NESTLER—Project: 101060762—HORIZON-CL6-2021-FARM2FORK-01), IKEA Foundation (G-2204-02144), the Rockefeller Foundation (WAVE-IN—Grant No. 2021 FOD 030); Bill & Melinda Gates Foundation (INV-032416); the Curt Bergfors Foundation Food Planet Prize Award; Norwegian Agency for Development Cooperation, the Section for research, innovation, and higher education grant number RAF–3058 KEN–18/0005 (CAP–Africa); Novo Nordic Foundation (RefIPro: NNF22SA0078466), the Swedish International Development Cooperation Agency (Sida); the Swiss Agency for Development and Cooperation (SDC); Australian Centre for International Agricultural Research (ACIAR), the Federal Democratic Republic of Ethiopia and the Government of the Republic of Kenya. EK was also financially supported by the Fogarty International Center of the National Institutes of Health (NIH) (Grant No. U2RTW010677) through the Eastern Africa Network for Bioinformatics Training (EANBiT). The funders had no role in study design, data collection and analysis, decision to publish, or preparation of the manuscript. The views expressed herein do not necessarily reflect the official opinion of the donors.

## Conflict of interest

The authors declare that the research was conducted in the absence of any commercial or financial relationships that could be construed as a potential conflict of interest.

## Publisher’s note

All claims expressed in this article are solely those of the authors and do not necessarily represent those of their affiliated organizations, or those of the publisher, the editors and the reviewers. Any product that may be evaluated in this article, or claim that may be made by its manufacturer, is not guaranteed or endorsed by the publisher.

## Footnotes

^1^ http://www.cazy.org/

^2^ https://github.com/nanoporetech/pychopper

^3^ https://ftp.ncbi.nlm.nih.gov/genomes/refseq/invertebrate/Hermetia_illucens/all_assembly_versions/GCF_905115235.1_iHerIll2.2.curated.20191125/

^4^ https://github.com/linnabrown/run_dbcan

^5^ https://bcb.unl.edu/dbCAN_PUL/blast
