# Supplementary material for: Metatranscriptomic analysis of the gut microbiome of black soldier fly larvae reared on lignocellulose-rich fiber diets unveils key lignocellulolytic enzymes
Source: Front Microbiol. 2023 Apr 26;14:1120224. doi: 10.3389/fmicb.2023.1120224 (PMC10171111; doi:10.3389/fmicb.2023.1120224)
Supplement: Supplementary file 1 [file Data_Sheet_1.docx]

Supplementary Material

Metatranscriptomic Analysis of the Gut Microbiome of Black Soldier Fly Larvae Bred on Lignocellulose-Rich Fiber Diets Unveils Key Lignocellulolytic Enzymes

Eric G. Kariuki^1,2*^, Caleb Kibet^1^, Juan C. Paredes^2^, Gerald Mboowa^2^, Oscar Mwaura^1^, John Njogu^1^, Daniel Masiga^1^, Timothy D.H. Bugg^3^ and Chrysantus M. Tanga^1*^

^1^International Centre of Insect Physiology and Ecology (*icipe*). P.O. Box 30772-00100, Nairobi, Kenya

^2^ Department of Immunology and Molecular Biology, Makerere University. P.O. Box 7062 Kampala, Uganda

^3^Department of Chemistry and School of Life Sciences, University of Warwick, Coventry CV4 7AL, United Kingdom

*** Correspondence:**

E.G. Kariuki; E-mail: [ericgathirwak@gmail.com](mailto:ericgathirwak@gmail.com); Tel.: +254 701049185

C. M. Tanga; E-mail: [ctanga@icipe.org](mailto:ctanga@icipe.org); Tel. : +254 702729931

# Supplementary Figures and Tables

## Supplementary Figures

**(A)**


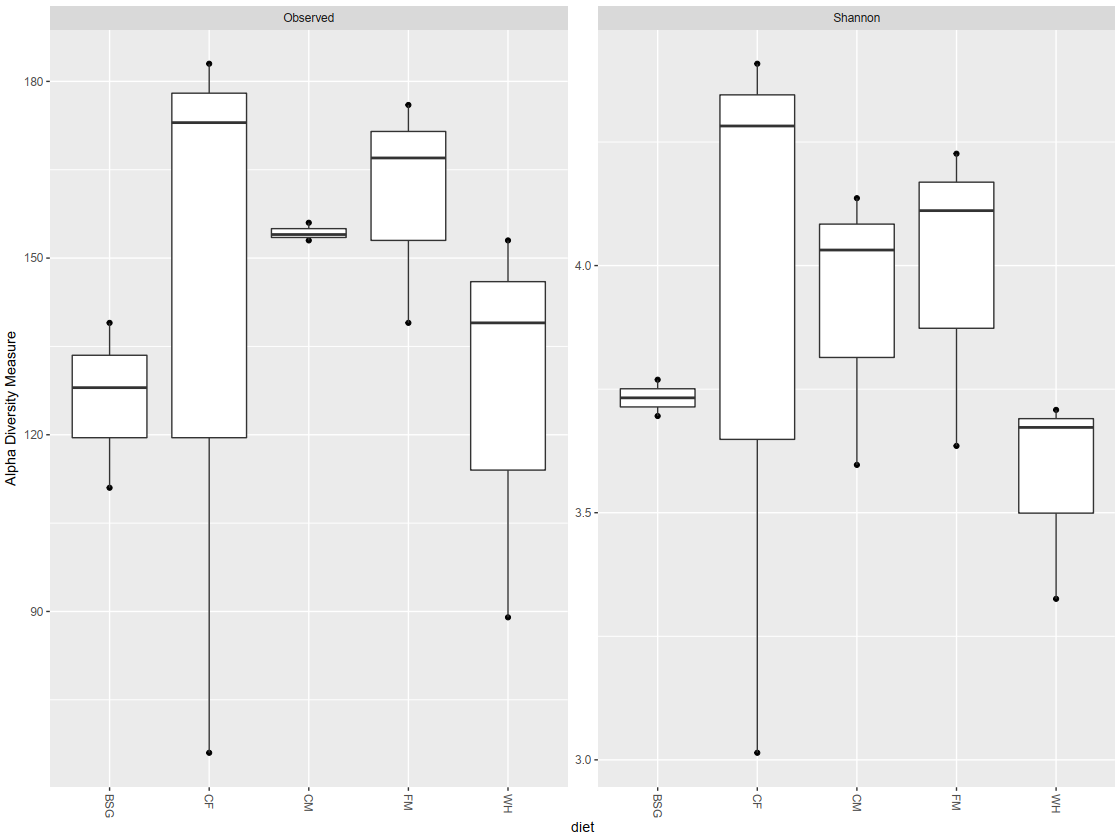


**(B)**

**
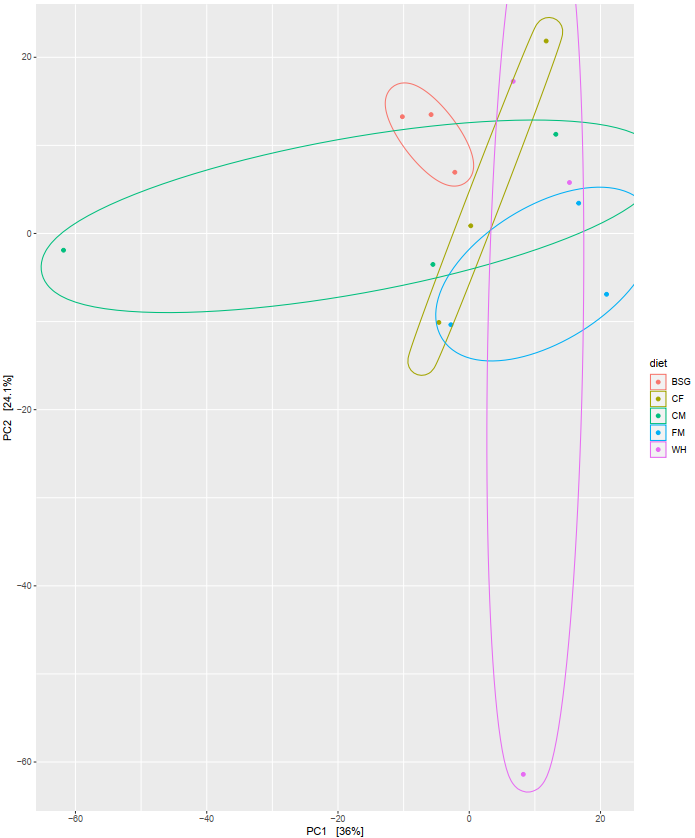
**

## Supplementary Figure 1.

Diversity statistics using ribodepleted 16S rRNA sequences. (A) Alpha Diversity: The highest observed diversity was recorded in the CF diet samples while the lowest observed diversity was recorded in the BSG diet samples. However, the lowest Shannon diversity was observed in the WH diet samples while the highest Shannon diversity was recorded in the CF diet samples. (B) Beta Diversity: The Bray-Curtis PCA plot recommended for dissimilarity ordination on organism abundance data showed that the two main principal components PC1 and PC2 accounted for 60.1% of the total variance with no distinct inter-dietary sample variance patterns observed.


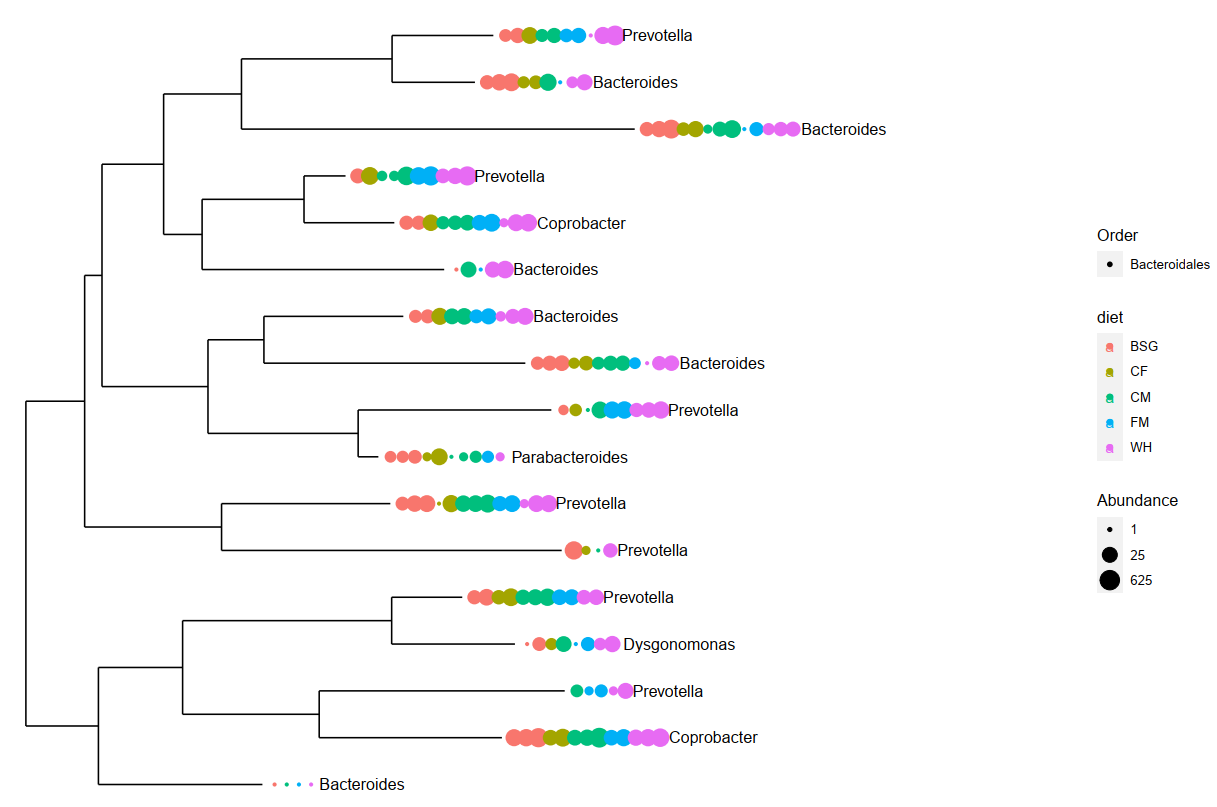


## Supplementary Figure 2.

Phylogenetic tree of 16S rRNA samples from order Bacteroidales. This figure shows the taxonomic relationship between the 5 abundant genera observed during 16S rRNA analysis in order Bacteroidales in the different dietary samples. The color of the dot represents the dietary sample, the number of dots in each clade represents the number of individual ASVs observed in each taxon, and the size of the dot indicates the abundance of individual ASVs in each taxon. There was a notable abundance of genus *Coprobacter* across all dietary substrates, a genus that was not observed in abundance from the Metatranscriptomics analysis.

## Supplementary Tables

## Supplementary Table 1.

Residual rRNA from the metatranscriptomes ranged from 25.6% to 44%. Sample CF1 recorded the highest number of unmapped reads (149,255) while sample WH2 recorded the lowest number of unmapped reads (2,716). The unmapped reads ranged from 4.04% (WH2) to 18.53% (FM3) of the total classified reads. The subsequent annotation steps were performed on these unmapped reads, while statistical analysis steps in R required these read counts to perform library normalization and differential statistics between the sample metatranscriptomes.

| ***Sample_ID*** | ***Total classified reads*** | ***No. of rRNA reads (%)*** | ***No. of unmapped reads (%)*** |
| --- | --- | --- | --- |
| *CF1* | 903,473 | 269,988 (30.40) | 149,255 (16.52) |
| *CF2* | 56,024 | 23,679 (44) | 3,747 (6.69) |
| *CF3* | 170,007 | 65,046 (39.10) | 18,073 (10.63) |
| *CF4* | 144,949 | 51,592 (36.20) | 12,021 (8.29) |
| *BSG1* | 953,056 | 243,009 (25.60) | 105,398 (11.06) |
| *BSG2* | 181,499 | 48,838 (27.90) | 21,247 (11.71) |
| *BSG3* | 101,893 | 35,883 (35.80) | 5,567 (5.46) |
| *CM1* | 803,584 | 238,871 (30.30) | 142,086 (17.68) |
| *CM2* | 170,876 | 44,796 (32.70) | 8,746 (5.12) |
| *CM3* | 251,968 | 79,060 (32.10) | 25,897 (10.28) |
| *FM1* | 691,444 | 196,270 (28.60) | 92,612 (13.39) |
| *FM2* | 96,446 | 38,193 (40.30) | 7,765 (8.05) |
| *FM3* | 296,918 | 103,445 (36.10) | 55,030 (18.53) |
| *WH1* | 884,170 | 271,258 (30.90) | 87,292 (9.87) |
| *WH2* | 67,176 | 28,313 (43.40) | 2,716 (4.04) |
| *WH3* | 170,605 | 62,809 (37.20) | 9,921 (5.82) |

## Supplementary table 2.

**Total number of unmapped reads per metatranscriptome**. From **Supplementary table 2**, sample CF1 recorded the highest number of unmapped reads (149,255) while sample WH2 recorded the lowest number of unmapped reads (2,716). The unmapped reads ranged from 4.04% (WH2) to 18.53% (FM3) of the total classified reads.

| ***Sample_ID*** | ***Total classified reads*** | ***No. of unmapped reads (%)*** |
| --- | --- | --- |
| *CF1* | 903,473 | 149,255 (16.52) |
| *CF2* | 56,024 | 3,747 (6.69) |
| *CF3* | 170,007 | 18,073 (10.63) |
| *CF4* | 144,949 | 12,021 (8.29) |
| *BSG1* | 953,056 | 105,398 (11.06) |
| *BSG2* | 181,499 | 21,247 (11.71) |
| *BSG3* | 101,893 | 5,567 (5.46) |
| *CM1* | 803,584 | 142,086 (17.68) |
| *CM2* | 170,876 | 8,746 (5.12) |
| *CM3* | 251,968 | 25,897 (10.28) |
| *FM1* | 691,444 | 92,612 (13.39) |
| *FM2* | 96,446 | 7,765 (8.05) |
| *FM3* | 296,918 | 55,030 (18.53) |
| *WH1* | 884,170 | 87,292 (9.87) |
| *WH2* | 67,176 | 2,716 (4.04) |
| *WH3* | 170,605 | 9,921 (5.82) |

## Supplementary Table 3.

This table shows the comparison between reads subjected to de novo clustering with isONclust (Sahlin & Medvedev, 2020) and correction with isONcorrect (Sahlin et al., 2021) and uncorrected reads that were neither clustered nor corrected. There was a slight variation in the mean coverage, mean depth, and mapping quality between the corrected and uncorrected reads. However, all the corrected reads recorded a higher (%) of reads that mapped to the BSF genome as compared to the uncorrected reads.

| ***Barcode_ID*** | ***Sample_ID*** | ***Mean coverage*** | | ***Mean depth*** | | ***Mapping quality*** | | ***(%) of mapped reads*** | |
| --- | --- | --- | --- | --- | --- | --- | --- | --- | --- |
|  |  | *cor.* | *uncor.* | *cor.* | *uncor.* | *cor.* | *uncor.* | *cor.* | *uncor.* |
| ***barcode01*** | CF1 | 0.262 | 0.264 | 0.043 | 0.052 | 52.96 | 52.33 | 94.55 | 93.66 |
| ***barcode02*** | FM1 | 0.373 | 0.373 | 0.015 | 0.015 | 52.5 | 52.84 | 90.93 | 86.75 |
| ***barcode03*** | BSG1 | 0.268 | 0.269 | 0.025 | 0.025 | 50.43 | 50.41 | 92.03 | 89.01 |
| ***barcode04*** | CM1 | 0.3 | 0.3 | 0.018 | 0.018 | 54.01 | 53.97 | 95.45 | 92.24 |
| ***barcode06*** | WH1 | 0.559 | 0.561 | 0.058 | 0.057 | 51.64 | 51.97 | 89.62 | 85.84 |
| ***barcode07*** | CM2 | 0.389 | 0.389 | 0.047 | 0.047 | 53.21 | 52.99 | 89.35 | 86.26 |
| ***barcode08*** | CF2 | 0.351 | 0.353 | 0.078 | 0.077 | 50.51 | 50.83 | 92.2 | 88.4 |
| ***barcode09*** | FM2 | 0.312 | 0.314 | 0.056 | 0.055 | 47.24 | 47.79 | 82.5 | 76.16 |
| ***barcode10*** | WH2 | 0.398 | 0.4 | 0.052 | 0.052 | 52 | 52.08 | 94.76 | 92.48 |
| ***barcode11*** | BSG2 | 0.43 | 0.43 | 0.035 | 0.034 | 53.03 | 53.08 | 95.09 | 92.75 |
| ***barcode12*** | CF3 | 0.406 | 0.407 | 0.0402 | 0.04 | 52.96 | 52.91 | 91.73 | 88.7 |
| ***barcode07_1*** | CM3 | 0.717 | 0.717 | 0.203 | 0.199 | 49.97 | 50.11 | 84.86 | 80.88 |
| ***barcode08_1*** | FM3 | 0.672 | 0.675 | 0.174 | 0.171 | 48.24 | 48.47 | 89.57 | 86.29 |
| ***barcode09_1*** | WH3 | 1.075 | 1.077 | 0.229 | 0.224 | 48.87 | 48.19 | 89.15 | 85.18 |
| ***barcode10_1*** | BSG3 | 1.09 | 1.094 | 0.162 | 0.158 | 47.71 | 48.2 | 83.3 | 78.06 |
| ***barcode11_1*** | CF4 | 0.572 | 0.574 | 0.223 | 0.22 | 49.59 | 49.44 | 92.42 | 90.17 |

**cor.*- corrected

**uncor*.-uncorrected

**Supplementary table 4.**

This table shows the metabolism of the most abundant organisms in the pooled metatranscriptomes.

| ***Diet*** | ***Organism (>0.1% Abundance)*** | ***Raw counts*** | ***Metabolism*** | ***(%)*** |
| --- | --- | --- | --- | --- |
| ***BSG*** | *Sphingobacterium sp.* | 723 | Aerobic (Brady & Marcon, 2008) | 4.529 |
|  | *Bacteroides sp.* | 283 | Anaerobic (Gajdács & Urbán, 2020) | 1.773 |
|  | *Dysgonomonas sp.* | 242 | Facultative anaerobic (Hironaga et al., 2008) | 1.516 |
|  | *Prevotella sp.* | 220 | Anaerobic (Gajdács & Urbán, 2020) | 1.377 |
|  | *Sphingobacterium spiritivorum* | 160 | Aerobic (Brady & Marcon, 2008) | 1.002 |
|  | *Dysgonomonas gadei* | 144 | Facultative anaerobic (Hironaga et al., 2008) | 0.901 |
|  | *Dysgonomonas mossii* | 136 | Facultative anaerobic (Hironaga et al., 2008) | 0.851 |
|  | *Bacteroidales* | 125 | Anaerobic (Berman, 2019) | 0.782 |
|  | *Sphingobacterium nematocida* | 98 | Aerobic (Brady & Marcon, 2008) | 0.613 |
|  | *Sphingobacteriaceae bacterium* | 97 | Aerobic (Brady & Marcon, 2008) | 0.607 |
|  | *Sphingobacterium deserti* | 93 | Aerobic (Brady & Marcon, 2008) | 0.583 |
|  | *Sphingobacterium thalpophilum* | 91 | Aerobic (Brady & Marcon, 2008) | 0.57 |
|  | *Sphingobacterium psychroaquaticum* | 90 | Aerobic (Brady & Marcon, 2008) | 0.564 |
|  | *Sphingobacterium paucimobilis* | 88 | Aerobic (Brady & Marcon, 2008) | 0.551 |
|  | *Dysgonomonas capnocytophagoides* | 82 | Facultative anaerobic (Hironaga et al., 2008) | 0.514 |
|  | *Dysgonomonas macrotermitis* | 79 | Facultative anaerobic (Hironaga et al., 2008) | 0.495 |
|  | *Bacteroides fragilis* | 56 | Anaerobic (Gajdács & Urbán, 2020) | 0.351 |
|  | *Bacteroidales bacterium* | 44 | Anaerobic (Gajdács & Urbán, 2020) | 0.275 |
|  | *Bacteroides thetaiotaomicron* | 41 | Anaerobic (Gajdács & Urbán, 2020) | 0.257 |
|  | *Prevotella ruminicola* | 25 | Anaerobic (Gajdács & Urbán, 2020) | 0.157 |
|  | *Bacteroides coprocola* | 22 | Anaerobic (Gajdács & Urbán, 2020) | 0.138 |
|  | *Bacteroides cellulosilyticus* | 21 | Anaerobic (Gajdács & Urbán, 2020) | 0.132 |
|  | *Bacteroides coprophilus* | 21 | Anaerobic (Gajdács & Urbán, 2020) | 0.132 |
|  | *Bacteroides luti* | 21 | Anaerobic (Gajdács & Urbán, 2020) | 0.132 |
|  | *Bacteroides clarus* | 20 | Anaerobic (Gajdács & Urbán, 2020) | 0.125 |
|  | *Bacteroides pyogenes* | 19 | Anaerobic (Gajdács & Urbán, 2020) | 0.12 |
|  | *Bacteroides finegoldii* | 19 | Anaerobic (Gajdács & Urbán, 2020) | 0.12 |
|  | *Bacteroides xylanisolvens* | 18 | Anaerobic (Gajdács & Urbán, 2020) | 0.112 |
|  | *Bacteroides uniformis* | 17 | Anaerobic (Gajdács & Urbán, 2020) | 0.106 |
|  | *Bacteroides coprosuis* | 17 | Anaerobic (Gajdács & Urbán, 2020) | 0.106 |
|  | *Bacteroides propionicifaciens* | 17 | Anaerobic (Gajdács & Urbán, 2020) | 0.106 |
|  | *Bacteroides vulgatus* | 16 | Anaerobic (Gajdács & Urbán, 2020) | 0.1 |
|  | *Bacteroides eggerthii* | 16 | Anaerobic (Gajdács & Urbán, 2020) | 0.1 |
| ***CF*** | *Sphingobacterium sp.* | 13250 | Aerobic (Brady & Marcon, 2008) | 10.14 |
|  | *Flavobacterium sp.* | 3695 | Aerobic/facultative anaerobic (Berman, 2019) | 2.828 |
|  | *Pedobacter sp.* | 2758 | Aerobic (Steyn et al., 1998) | 2.111 |
|  | *Sphingobacterium spiritivorum* | 2348 | Aerobic (Brady & Marcon, 2008) | 1.797 |
|  | *Myroides sp.* | 2094 | Aerobic (Jooste & Hugo, 1999) | 1.603 |
|  | *Sphingobacterium deserti* | 1653 | Aerobic (Brady & Marcon, 2008) | 1.265 |
|  | *Myroides oratus* | 1644 | Aerobic (Jooste & Hugo, 1999) | 1.259 |
|  | *Sphingobacterium psychroaquaticum* | 1560 | Aerobic (Brady & Marcon, 2008) | 1.194 |
|  | *Sphingobacterium nematocida* | 1558 | Aerobic (Brady & Marcon, 2008) | 1.193 |
|  | *Sphingobacteriaceae bacterium* | 1521 | Aerobic (Brady & Marcon, 2008) | 1.164 |
|  | *Sphingobacterium paucimobilis* | 1447 | Aerobic (Brady & Marcon, 2008) | 1.108 |
|  | *Sphingobacterium thalpophilum* | 1423 | Aerobic (Brady & Marcon, 2008) | 1.089 |
|  | *Myroides odoratimimus* | 896 | Aerobic (Jooste & Hugo, 1999) | 0.686 |
|  | *Myroides injenensis* | 837 | Aerobic (Jooste & Hugo, 1999) | 0.641 |
|  | *Flavobacterium columnare* | 835 | Aerobic/facultative anaerobic (Berman, 2019) | 0.639 |
|  | *Pedobacter glucosidilyticus* | 655 | Aerobic (Steyn et al., 1998) | 0.501 |
|  | *Myroides marinus* | 492 | Aerobic (Jooste & Hugo, 1999) | 0.377 |
|  | *Flavobacterium suncheonense* | 391 | Aerobic/facultative anaerobic (Berman, 2019) | 0.299 |
|  | *Myroides profundi* | 389 | Aerobic (Jooste & Hugo, 1999) | 0.298 |
|  | *Pedobacter luteus* | 372 | Aerobic (Steyn et al., 1998) | 0.285 |
|  | *Pedobacter oryzae* | 366 | Aerobic (Steyn et al., 1998) | 0.28 |
|  | *Flavobacteria bacterium* | 340 | Aerobic/facultative anaerobic (Berman, 2019) | 0.26 |
|  | *Flavobacterium beibuense* | 337 | Aerobic/facultative anaerobic (Berman, 2019) | 0.258 |
|  | *Pedobacter arcticus* | 315 | Aerobic (Steyn et al., 1998) | 0.241 |
|  | *Flavobacterium enshiense* | 306 | Aerobic/facultative anaerobic (Berman, 2019) | 0.234 |
|  | *Flavobacterium haoranii* | 292 | Aerobic/facultative anaerobic (Berman, 2019) | 0.224 |
|  | *Pedobacter steynii* | 283 | Aerobic (Steyn et al., 1998) | 0.217 |
|  | *Flavobacterium terrae* | 282 | Aerobic/facultative anaerobic (Berman, 2019) | 0.216 |
|  | *Flavobacterium indicum* | 272 | Aerobic/facultative anaerobic (Berman, 2019) | 0.208 |
|  | *Flavobacterium cauense* | 258 | Aerobic/facultative anaerobic (Berman, 2019) | 0.197 |
|  | *Flavobacterium fontis* | 257 | Aerobic/facultative anaerobic (Berman, 2019) | 0.197 |
|  | *Flavobacterium saliperosum* | 250 | Aerobic/facultative anaerobic (Berman, 2019) | 0.191 |
|  | *Flavobacterium filum* | 236 | Aerobic/facultative anaerobic (Berman, 2019) | 0.181 |
|  | *Flavobacterium cucumis* | 235 | Aerobic/facultative anaerobic (Berman, 2019) | 0.18 |
|  | *Flavobacterium psychrophilum* | 234 | Aerobic/facultative anaerobic (Berman, 2019) | 0.179 |
|  | *Pedobacter africanus* | 229 | Aerobic (Steyn et al., 1998) | 0.175 |
|  | *Flavobacterium sasangense* | 229 | Aerobic/facultative anaerobic (Berman, 2019) | 0.175 |
|  | *Flavobacterium akiainvivens* | 229 | Aerobic/facultative anaerobic (Berman, 2019) | 0.175 |
|  | *Flavobacterium limnosediminis* | 229 | Aerobic/facultative anaerobic (Berman, 2019) | 0.175 |
|  | *Flavobacterium subsaxonicum* | 220 | Aerobic/facultative anaerobic (Berman, 2019) | 0.168 |
|  | *Pedobacter antarcticus* | 217 | Aerobic (Steyn et al., 1998) | 0.166 |
|  | *Flavobacterium rivuli* | 205 | Aerobic/facultative anaerobic (Berman, 2019) | 0.16 |
|  | *Flavobacterium johnsoniae* | 202 | Aerobic/facultative anaerobic (Berman, 2019) | 0.155 |
|  | *Pedobacter nyackensis* | 200 | Aerobic (Steyn et al., 1998) | 0.153 |
|  | *Flavobacterium gelidilacus* | 195 | Aerobic/facultative anaerobic (Berman, 2019) | 0.149 |
|  | *Pedobacter cryoconitis* | 189 | Aerobic (Steyn et al., 1998) | 0.145 |
|  | *Flavobacterium frigoris* | 188 | Aerobic/facultative anaerobic (Berman, 2019) | 0.145 |
|  | *Pedobacter panaciterrae* | 181 | Aerobic (Steyn et al., 1998) | 0.139 |
|  | *Pedobacter heparinus* | 178 | Aerobic (Steyn et al., 1998) | 0.136 |
|  | *Flavobacterium daejeonense* | 167 | Aerobic/facultative anaerobic (Berman, 2019) | 0.128 |
|  | *Flavobacterium branchiophilum* | 162 | Aerobic/facultative anaerobic (Berman, 2019) | 0.124 |
|  | *Flavobacterium soli* | 159 | Aerobic/facultative anaerobic (Berman, 2019) | 0.122 |
|  | *Pedobacter jeongneungensis* | 158 | Aerobic (Steyn et al., 1998) | 0.121 |
|  | *Pedobacter kyungheensis* | 155 | Aerobic (Steyn et al., 1998) | 0.118 |
|  | *Flavobacterium aquatile* | 150 | Aerobic/facultative anaerobic (Berman, 2019) | 0.115 |
|  | *Pedobacter lusitanus* | 145 | Aerobic (Steyn et al., 1998) | 0.111 |
|  | *Pedobacter caeni* | 145 | Aerobic (Steyn et al., 1998) | 0.111 |
|  | *Flavobacterium antarcticum* | 145 | Aerobic/facultative anaerobic (Berman, 2019) | 0.111 |
|  | *Pedobacter agri* | 140 | Aerobic (Steyn et al., 1998) | 0.107 |
|  | *Flavobacterium flevense* | 137 | Aerobic/facultative anaerobic (Berman, 2019) | 0.105 |
|  | *Flavobacterium succinicans* | 136 | Aerobic/facultative anaerobic (Berman, 2019) | 0.104 |
|  | *Flavobacterium fluvii* | 136 | Aerobic/facultative anaerobic (Berman, 2019) | 0.104 |
| ***CM*** | *Sphingobacterium sp.* | 3182 | Aerobic (Brady & Marcon, 2008) | 2.936 |
|  | *Clostridium sp.* | 1385 | Anaerobic (Wells & Wilkins, 1996) | 1.278 |
|  | *Bacillus sp.* | 1353 | Aerobic (Minnaard, Rolny & Pérez, 1996) | 1.249 |
|  | *Chryseobacterium sp.* | 1277 | Aerobic (Jooste & Hugo, 1999) | 1.179 |
|  | *Sphingobacterium spiritivorum* | 572 | Aerobic (Brady & Marcon, 2008) | 0.528 |
|  | *Clostridium botulinum* | 474 | Anaerobic (Wells & Wilkins, 1996) | 0.438 |
|  | *Clostridioides difficile* | 458 | Anaerobic (Wells & Wilkins, 1996) | 0.423 |
|  | *Sphingobacterium nematocida* | 387 | Aerobic (Brady & Marcon, 2008) | 0.358 |
|  | *Sphingobacterium paucimobilis* | 383 | Aerobic (Brady & Marcon, 2008) | 0.353 |
|  | *Bacillus cereus* | 377 | Aerobic or facultatively anaerobic (Bottone, 2010) | 0.348 |
|  | *Sphingobacterium psychroaquaticum* | 376 | Aerobic (Brady & Marcon, 2008) | 0.347 |
|  | *Sphingobacterium deserti* | 353 | Aerobic (Brady & Marcon, 2008) | 0.326 |
|  | *Sphingobacterium thalpophilum* | 324 | Aerobic (Brady & Marcon, 2008) | 0.299 |
|  | *Clostridium ultunense* | 169 | Anaerobic (Wells & Wilkins, 1996) | 0.156 |
|  | *Clostridium purinilyticum* | 157 | Anaerobic (Wells & Wilkins, 1996) | 0.145 |
|  | *Clostridium perfringens* | 141 | Anaerobic (Wells & Wilkins, 1996) | 0.13 |
|  | *Clostridium formicaceticum* | 120 | Anaerobic (Wells & Wilkins, 1996) | 0.111 |
|  | *Bacillus megaterium* | 111 | Aerobic (Goswami et al., 2018) | 0.102 |
| ***FM*** | *Sphingobacterium sp.* | 7242 | Aerobic (Brady & Marcon, 2008) | 7.946 |
|  | *Flavobacterium sp.* | 2019 | Aerobic/facultative anaerobic (Berman, 2019) | 2.281 |
|  | *Pedobacter sp.* | 1712 | Aerobic (Steyn et al., 1998) | 1.94 |
|  | *Chryseobacterium sp.* | 1029 | Aerobic (Jooste & Hugo, 1999) | 1.166 |
|  | *Sphingobacterium deserti* | 876 | Aerobic (Brady & Marcon, 2008) | 0.993 |
|  | *Sphingobacterium psychroaquaticum* | 858 | Aerobic (Brady & Marcon, 2008) | 0.973 |
|  | *Sphingobacteriaceae bacterium* | 807 | Aerobic (Brady & Marcon, 2008) | 0.915 |
|  | *Sphingobacterium nematocida* | 794 | Aerobic (Brady & Marcon, 2008) | 0.9 |
|  | *Sphingobacterium paucimobilis* | 778 | Aerobic (Brady & Marcon, 2008) | 0.882 |
|  | *Sphingobacterium thalpophilum* | 774 | Aerobic (Brady & Marcon, 2008) | 0.878 |
|  | *Flavobacterium columnare* | 550 | Aerobic/facultative anaerobic (Berman, 2019) | 0.624 |
|  | *Pedobacter glucosidilyticus* | 513 | Aerobic (Steyn et al., 1998) | 0.582 |
|  | *Pedobacter luteus* | 352 | Aerobic (Steyn et al., 1998) | 0.399 |
|  | *Pedobacter oryzae* | 343 | Aerobic (Steyn et al., 1998) | 0.389 |
|  | *Pedobacter arcticus* | 338 | Aerobic (Steyn et al., 1998) | 0.383 |
|  | *Flavobacterium haoranii* | 189 | Aerobic/facultative anaerobic (Berman, 2019) | 0.214 |
|  | *Flavobacterium suncheonense* | 178 | Aerobic/facultative anaerobic (Berman, 2019) | 0.202 |
|  | *Flavobacterium terrae* | 171 | Aerobic/facultative anaerobic (Berman, 2019) | 0.194 |
|  | *Flavobacterium indicum* | 166 | Aerobic/facultative anaerobic (Berman, 2019) | 0.188 |
|  | *Flavobacterium psychrophilum* | 156 | Aerobic/facultative anaerobic (Berman, 2019) | 0.177 |
|  | *Flavobacterium filum* | 150 | Aerobic/facultative anaerobic (Berman, 2019) | 0.17 |
|  | *Flavobacterium beibuense* | 145 | Aerobic/facultative anaerobic (Berman, 2019) | 0.164 |
|  | *Pedobacter africanus* | 144 | Aerobic (Steyn et al., 1998) | 0.163 |
|  | *Pedobacter nyackensis* | 130 | Aerobic (Steyn et al., 1998) | 0.147 |
|  | *Flavobacterium enshiense* | 130 | Aerobic/facultative anaerobic (Berman, 2019) | 0.147 |
|  | *Flavobacterium cucumis* | 130 | Aerobic/facultative anaerobic (Berman, 2019) | 0.147 |
|  | *Flavobacterium akiainvivens* | 125 | Aerobic/facultative anaerobic (Berman, 2019) | 0.141 |
|  | *Flavobacterium cauense* | 117 | Aerobic/facultative anaerobic (Berman, 2019) | 0.132 |
|  | *Flavobacterium rivuli* | 114 | Aerobic/facultative anaerobic (Berman, 2019) | 0.129 |
|  | *Flavobacterium sasangense* | 113 | Aerobic/facultative anaerobic (Berman, 2019) | 0.128 |
|  | *Parabacteroides merdae* | 113 | Anaerobic (Brady & Marcon, 2008) | 0.128 |
|  | *Flavobacterium subsaxonicum* | 110 | Aerobic/facultative anaerobic (Berman, 2019) | 0.125 |
|  | *Pedobacter steynii* | 109 | Aerobic (Steyn et al., 1998) | 0.124 |
|  | *Flavobacterium johnsoniae* | 108 | Aerobic/facultative anaerobic (Berman, 2019) | 0.122 |
|  | *Flavobacterium branchiophilum* | 103 | Aerobic/facultative anaerobic (Berman, 2019) | 0.117 |
|  | *Flavobacterium saliperosum* | 98 | Aerobic/facultative anaerobic (Berman, 2019) | 0.111 |
|  | *Pedobacter heparinus* | 98 | Aerobic (Steyn et al., 1998) | 0.111 |
|  | *Flavobacterium frigoris* | 97 | Aerobic/facultative anaerobic (Berman, 2019) | 0.11 |
|  | *Pedobacter antarcticus* | 96 | Aerobic (Steyn et al., 1998) | 0.109 |
|  | *Flavobacterium daejeonense* | 94 | Aerobic/facultative anaerobic (Berman, 2019) | 0.107 |
|  | *Pedobacter panaciterrae* | 91 | Aerobic (Steyn et al., 1998) | 0.103 |
|  | *Flavobacterium flevense* | 90 | Aerobic/facultative anaerobic (Berman, 2019) | 0.102 |
|  | *Flavobacterium aquatile* | 90 | Aerobic/facultative anaerobic (Berman, 2019) | 0.102 |
|  | *Flavobacterium limnosediminis* | 89 | Aerobic/facultative anaerobic (Berman, 2019) | 0.101 |
|  | *Pedobacter cryoconitis* | 89 | Aerobic (Steyn et al., 1998) | 0.101 |
| ***WH*** | *Gilliamella apicola* | 831 | Microaerobic/anaerobic (Reimer et al., 2022) | 2.797 |
|  | *Parabacteroides sp.* | 580 | Anaerobic (Brady & Marcon, 2008) | 1.952 |
|  | *Bacteroides sp.* | 491 | Anaerobic (Gajdács & Urbán, 2020) | 1.649 |
|  | *Dysgonomonas sp.* | 433 | Facultative anaerobic (Hironaga et al., 2008) | 1.457 |
|  | *Dysgonomonas mossii* | 267 | Facultative anaerobic (Hironaga et al., 2008) | 0.899 |
|  | *Dysgonomonas gadei* | 244 | Facultative anaerobic (Hironaga et al., 2008) | 0.821 |
|  | *Gilliamella sp.* | 205 | Microaerobic/anaerobic (Zheng et al., 2019) | 0.69 |
|  | *Dysgonomonas capnocytophagoides* | 180 | Facultative anaerobic (Hironaga et al., 2008) | 0.606 |
|  | *Dysgonomonas macrotermitis* | 170 | Facultative anaerobic (Hironaga et al., 2008) | 0.572 |
|  | *Parabacteroides distasonis* | 142 | Anaerobic (Gajdács & Urbán, 2020) | 0.478 |
|  | *Bacteroides fragilis* | 96 | Anaerobic (Gajdács & Urbán, 2020) | 0.323 |
|  | *Parabacteroides merdae* | 91 | Anaerobic (Gajdács & Urbán, 2020) | 0.306 |
|  | *Parabacteroides chartae* | 78 | Anaerobic (Gajdács & Urbán, 2020) | 0.263 |
|  | *Bacteroides thetaiotaomicron* | 67 | Anaerobic (Gajdács & Urbán, 2020) | 0.226 |
|  | *Parabacteroides johnsonii* | 64 | Anaerobic (Gajdács & Urbán, 2020) | 0.215 |
|  | *Parabacteroides goldsteinii* | 62 | Anaerobic (Gajdács & Urbán, 2020) | 0.209 |
|  | *Bacteroides uniformis* | 50 | Anaerobic (Gajdács & Urbán, 2020) | 0.168 |
|  | *Bacteroides finegoldii* | 41 | Anaerobic (Gajdács & Urbán, 2020) | 0.138 |
|  | *Bacteroides pyogenes* | 41 | Anaerobic (Gajdács & Urbán, 2020) | 0.138 |
|  | *Bacteroides massiliensis* | 39 | Anaerobic (Gajdács & Urbán, 2020) | 0.131 |
|  | *Bacteroides ovatus* | 39 | Anaerobic (Gajdács & Urbán, 2020) | 0.131 |
|  | *Gilliamella mensalis* | 33 | Microaerobic/anaerobic (Zheng et al., 2019) | 0.111 |
|  | *Bacteroides vulgatus* | 31 | Anaerobic (Gajdács & Urbán, 2020) | 0.104 |
